# Structural basis for the recognition of two consecutive mutually interacting DPF motifs by the SGIP1 μ homology domain

**DOI:** 10.1038/srep19565

**Published:** 2016-01-29

**Authors:** Atsushi Shimada, Atsuko Yamaguchi, Daisuke Kohda

**Affiliations:** 1 Division of Structural Biology, Medical Institute of Bioregulation, Kyushu University, 3-1-1 Maidashi, Higashi-ku, Fukuoka 812-8582, Japan; 2 RIKEN Structural Biology Laboratory, 1-7-22 Suehiro-cho, Tsurumi, Yokohama 230-0045, Japan

## Abstract

FCHo1, FCHo2, and SGIP1 are key regulators of clathrin-mediated endocytosis. Their μ homology domains (μHDs) interact with the C-terminal region of an endocytic scaffold protein, Eps15, containing fifteen Asp-Pro-Phe (DPF) motifs. Here, we show that the high-affinity μHD-binding site in Eps15 is a region encompassing six consecutive DPF motifs, while the minimal μHD-binding unit is two consecutive DPF motifs. We present the crystal structures of the SGIP1 μHD in complex with peptides containing two DPF motifs. The peptides bind to a novel ligand-binding site of the μHD, which is distinct from those of other distantly related μHD-containing proteins. The two DPF motifs, which adopt three-dimensional structures stabilized by sequence-specific intramotif and intermotif interactions, are extensively recognized by the μHD and are both required for binding. Thus, consecutive and singly scattered DPF motifs play distinct roles in μHD binding.

Clathrin-mediated endocytosis (CME) is a process by which eukaryotic cells internalize extracellular molecules. It plays a critical role in numerous physiological phenomena, such as cell surface receptor internalization, nutrient uptake, and neurotransmission, and is exploited by viruses and bacteria for their entry into cells^1^. Many proteins involved in CME contain repeated sequence motifs, such as the Asp-Pro-Phe (DPF), Asn-Pro-Phe (NPF), and Asp-Pro-Trp (DPW) motifs^2^,^3^. The repetition of motifs is likely to play a critical role in the functions of these proteins, but the physiological meanings remain elusive.

Epidermal growth factor (EGF) pathway substrate 15 (Eps15) is involved in the clathrin assembly step of CME, and contains three Eps15 homology (EH) domains in the N-terminal region and a predicted unstructured region with fifteen DPF motifs in the C-terminal region^4^,^5^ (Fig. 1a). One of the best-characterized binding partners of the Eps15 DPF motifs is the α-adaptin appendage domain of the adaptor protein 2 (AP-2) complex^3^,^6^,^7^. Recently, FER-CIP4 homology (FCH) domain only 1 (FCHo1) and FCHo2 were also shown to bind to the DPF motif-rich region of Eps15 through their μ homology domains (μHDs), which share weak homology with the μ subunits of the adaptor protein complexes, such as AP-2^8^,^9^,^10^. The DPF motifs of another DPF motif-containing endocytic protein, Disabled-2 (Dab2), were reported to directly bind to the FCHo2 μHD^11^. However, the details of this interaction, such as the number of DPF motifs involved in binding, remained unclear.

The N-terminal regions of FCHo1/FCHo2 contain a lipid interacting module, the extended FCH (EFC)/FCH and BAR (F-BAR) domain, which interacts with the plasma membrane^12^,^13^,^14^,^15^,^16^ (Supplementary Fig. 1a). By interacting with Eps15 and the plasma membrane, FCHo1/FCHo2 recruit Eps15 to the plasma membrane^9^. Eps15 then acts as a scaffold to support the accumulation of the AP-2 complex on the plasma membrane, which facilitates clathrin assembly to initiate CME^9^,^17^.

Src homology 3 (SH3)-domain growth factor receptor-bound 2-like (endophilin) interacting protein 1 (SGIP1) and its splicing variant, SGIP1α, are brain-specific homologs of FCHo1/FCHo2^18^,^19^. The μHDs of SGIP1 and SGIP1α, which are identical to each other, are highly homologous to those of FCHo1/FCHo2 (Supplementary Fig. 1b). The μHD of SGIP1/SGIP1α also binds to the DPF motif-rich region of Eps15^19^. SGIP1α contains a lipid-binding domain called the membrane phospholipid-binding (MP) domain, instead of the EFC/F-BAR domain, in its N-terminal region^19^, while SGIP1 contains only a partial MP domain (Supplementary Fig. 1a). Quite recent reports revealed that FCHo1/FCHo2 and SGIP1 contain a conserved AP-2 complex activation motif in a largely unstructured linker region between the EFC/F-BAR domains and the partial MP domain, respectively, and their μHDs^20^,^21^. This finding further emphasizes the critical role of these proteins in CME.

Here, we identified the high- and low-affinity binding sites of the SGIP1 μHD in Eps15. The high-affinity μHD-binding site is composed of six consecutive DPF motifs connected by 2–3 residue linkers, while the low-affinity binding site is formed by two consecutive DPF motifs connected by a 5-residue linker. The minimum requirement for μHD binding comprised two consecutive DPF motifs, connected by a short and presumably flexible linker. We determined the crystal structures of the complexes between the SGIP1 μHD and the Eps15-derived peptides containing two consecutive DPF motifs. In the structures, the two consecutive DPF motifs adopt an ordered structure stabilized by intramotif and intermotif interactions, which are specifically recognized by the conserved recognition cleft of the μHD. Thus, the SGIP1/FCHo1/FCHo2 μHD is a domain designed for recognizing the locally ordered structure formed by the two consecutive DPF motifs. This finding demonstrates that the DPF motifs scattered in a single polypeptide are not functionally equivalent, and confirms that the consecutive DPF motifs play a distinct role from those of the other DPF motifs in μHD binding, and thus in CME.

## Results

### Identification of the SGIP1 μHD-binding sites in Eps15

**Figure 1:**
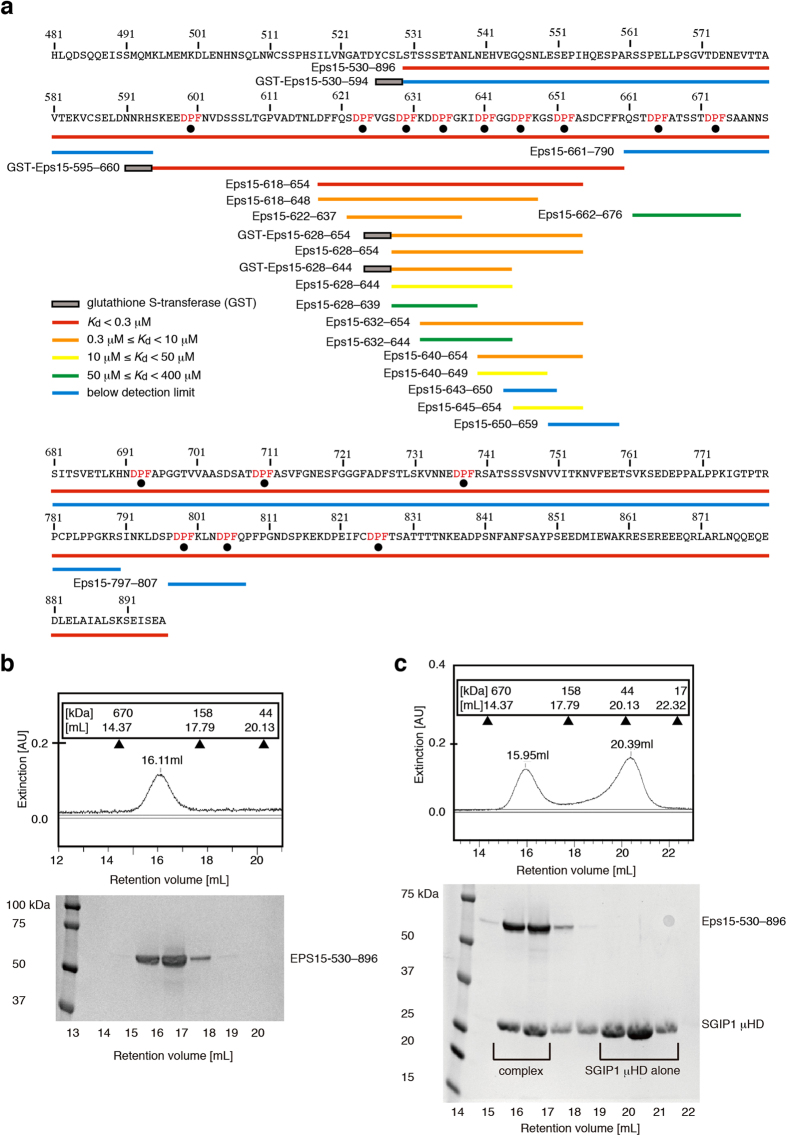
Identification of the SGIP1 μHD-binding sites in Eps15. (**a**) Eps15 fragments used for analytical gel filtration and ITC experiments. The amino acid sequence of the C-terminal region of Eps15 is shown, with the DPF motifs colored red and indicated by black dots. Eps15 fragments used for analytical gel filtration and ITC experiments are indicated as bars below the corresponding regions of the amino acid sequence of Eps15. The bars are colored red, orange, yellow, green, and cyan, according to the binding strength of the corresponding fragments to the μHD. The fragments are labeled according to the fragment names in Supplementary Tables 1 and 2. (**b**) The SDS-PAGE gel pattern of the elution fractions from the gel filtration analysis of Eps15-530–896. The Superdex 200 elution profile and the SDS-PAGE analysis of the fractions revealed that the apparent molecular weight of Eps15-530–896 deduced from the elution volume is significantly higher than the true molecular weight. (**c**) The SDS-PAGE gel pattern of the elution fractions from gel filtration, showing the equimolar binding of Eps15-530–896 and the SGIP1 μHD. The Superdex 200 elution profile and the SDS-PAGE analysis of the fractions demonstrated that one peak corresponds to the complex of Eps15-530–896 and the SGIP1 μHD, and the other peak corresponds to the SGIP1 μHD alone.

To gain insights into the mechanism of Eps15 recognition by the μHD, we set out to identify the μHD-binding sites in Eps15. We first performed analytical gel filtration experiments using an Eps15 fragment spanning residues 530 to 896 (Eps15-530–896; Fig. 1a and Supplementary Table 1). The apparent molecular weight of Eps15-530–896 deduced from analytical gel filtration (~340 kDa) significantly deviated from the true molecular weight of Eps15-530–896 (~40 kDa), probably due to its unstructured nature or oligomerization (Fig. 1b).

We next analyzed the mixture of Eps15-530–896 and the SGIP1 μHD (residues 552 to 828), mixed in a 1:2.4 molar ratio, by gel filtration (Fig. 1c). The chromatogram of this experiment clearly showed two peaks. One of them corresponded to the complex of Eps15-530–896 and the μHD, and the other peak corresponded to the μHD alone. The ratio of the Eps15-530–896-bound μHD to the unbound μHD indicated the equimolar binding of the μHD to Eps15-530–896. The apparent molecular weight of the complex of Eps15-530–896 and the μHD deduced from the analytical gel filtration was ~370 kDa. As μHD binding to Eps15-530–896 may convert Eps15-530–896 into a more compact form and thus reduce the apparent molecular weight of the complex, we could not determine whether the complex is a 1:1 complex or a higher-order oligomer, such as a 2:2 complex. In any case, these data clearly demonstrate that there is only one high-affinity μHD-binding site in the Eps15-530–896 molecule.

To confirm this conclusion, we performed an isothermal titration calorimetry (ITC) analysis of the interaction between Eps15-530–896 and the SGIP1 μHD. Since the ITC data did not fit the 1:1 binding curve reasonably well, we analyzed the data with the two-site model (Supplementary Fig. 2a–e and Experiment 1 in Supplementary Table 2). The data analysis indicated that there is indeed one high-affinity μHD-binding site in Eps15-530–896, which binds to the μHD with a dissociation constant (*K*_d_) of ~130 nM, and at least one additional weaker μHD-binding site (Supplementary Fig. 2a–e and Supplementary Table 2). Although the data were fitted with the two-site model, we realized that the data could be fitted equally well with the three-site model, as it contains more parameters. Thus, the total number of weak binding sites in Eps15 presently remains as an open question.

We then prepared shorter Eps15 fragments and tested their ability to bind to the μHD by ITC (Fig. 1a, Supplementary Fig. 3 and Supplementary Tables 1 and 2). We found that an Eps15 fragment corresponding to a 37-residue region containing six consecutive DPF motifs (Eps15-618–654; Fig. 1a and Supplementary Table 1) possessed one high-affinity binding site for the μHD with a *K*_d_ of ~65 nM, a value comparable to that of the high-affinity binding site of Eps15-530–896 (Experiment 4 fitted with the two-site model in Supplementary Table 2). The ITC data also suggested that Eps15-618–654 might possess an additional weak μHD-binding site, because the data required the two-site model, rather than the single-site model, for a reasonable fit (Supplementary Fig. 2f–j and Experiment 4 fitted with the single- and two-site models in Supplementary Table 2). In contrast, the binding of an Eps15 fragment containing the first five DPF motifs of Eps15-618–654 (Eps15-618–648) to the μHD was significantly weaker than that of Eps15-618–654, with a *K*_d_ of ~0.4 μM. Similarly, the binding of an Eps15 fragment containing the last five DPF motifs of Eps15-618–654 (Eps15-628–654) to the μHD was even weaker than that of Eps15-618–648, with a *K*_d_ of ~1.1 μM. Thus, hereafter we refer to the 37-residue region corresponding to Eps15-618–654 as the high-affinity binding site, although this region may contain an additional weak-binding site as well as the true high-affinity binding site. A longer 66-residue region of Eps15 (residues 595 to 660), containing the entire region corresponding to Eps15-618–654, previously showed significant binding to the homologous FCHo1 μHD by pull-down assays^22^. This suggests that the μHDs of SGIP1 and FCHo1 bind to the same high-affinity binding site in Eps15.

**Figure 2:**
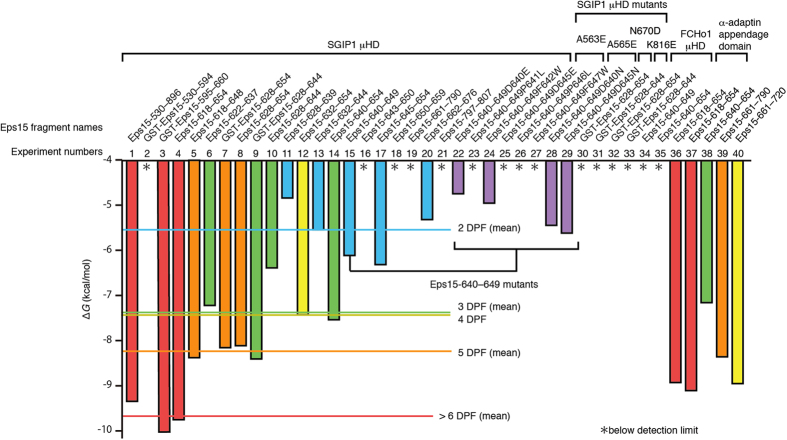
The Δ*G* values of the interactions between Eps15 fragments and various μHD and α-adaptin appendage domain samples, determined by ITC. The experiment numbers and Eps15 fragment names correspond to those in Supplementary Table 2. For the ITC experiments analyzed with the two-site model, only the Δ*G* values for the higher-affinity binding sites are shown. The bars are colored according to the numbers of DPF motifs in the Eps15 fragment used in each ITC experiment (0–1 DPF motifs: purple; 2 DPF motifs: cyan; 3 DPF motifs: green; 4 DPF motifs: yellow; 5 DPF motifs: orange; 6–15 DPF motifs: red). The mean Δ*G* values of the interactions between the SGIP1 μHD and respective Eps15 fragments containing the same numbers of consecutive DPF motifs are indicated, and the data below the detection limit were excluded from the calculation of the mean values. Larger negative values of Δ*G* indicate higher affinities between the two molecules.

In this previous report, a shorter Eps15 region containing the first three DPF motifs of the high-affinity binding site of Eps15 (residues from 595 to 636) also bound to the FCHo1 μHD, albeit with reduced affinity^22^. Consistently, an Eps15 fragment containing these first three DPF motifs (Eps15-622–637; Fig. 1a) bound to the SGIP1 μHD, but with significantly reduced affinity as compared with that of Eps15-530–896 (Figs 1a and 2 and Supplementary Table 2). Interestingly, an Eps15 fragment containing the last three DPF motifs of the high-affinity binding site (Eps15-640–654; Figs 1a and 2), which does not overlap with Eps15-622–637, also bound to the μHD with slightly stronger affinity than Eps15-622–637. Moreover, another Eps15 fragment containing the second, third, and fourth DPF motifs in the high-affinity binding site (Eps15-628–644; Fig. 1a) also bound to the μHD, but with significantly weaker affinity than Eps15-622–637 and Eps15-640–654. These results suggest that high-affinity binding requires all six of the consecutive DPF motifs, and there is no particular shorter region that is strictly required for μHD binding in the high-affinity binding site.

To identify the necessary conditions for modest μHD binding, we prepared Eps15 fragments containing only one or two DPF motifs, corresponding to different parts of Eps15-618–654, and tested their abilities to bind to the μHD (Figs 1a and 2, Supplementary Fig. 3 and Supplementary Tables 1 and 2). These experiments indicated that at least two consecutive DPF motifs are required for binding to the SGIP1 μHD. Comparisons of the strengths of the affinities of the μHD for various Eps15 fragments with different numbers of DPF motifs revealed that the Eps15 fragments with more DPF motifs tend to bind more strongly to the μHD (Figs 1a and 2 and Supplementary Table 2).

Two locations other than the high-affinity binding site in Eps15 contain two adjacent DPF motifs. Among the short fragments corresponding to these two regions, an Eps15 fragment spanning residues 662 to 676 (Eps15-662–676; Fig. 1a) weakly bound to the μHD, while the other Eps15 fragment spanning residues 797 to 807 (Eps15-797–807; Fig. 1a) did not (Figs 1a and 2 and Supplementary Table 2). Thus, we identified another relatively weak binding site located in close proximity to the high-affinity binding site, which corresponds to one of the additional weak binding sites in Eps15-530–896. Although Eps15-662–676 bound to the μHD, we could not detect the clear binding of a longer Eps15 fragment, containing the entire region corresponding to Eps15-662–676, to the μHD (Eps15-661–790 in Fig. 1a). This is probably due to the insufficient concentrations of proteins used in the corresponding ITC experiments, because of sample limitations, and the quality of the obtained data (Experiment 19 in Supplementary Fig. 3 and Supplementary Table 2). The reason why Eps15-797–807 did not bind to the μHD will be discussed in a later section.

### Crystal structures of the SGIP1 μHD

**Figure 3:**
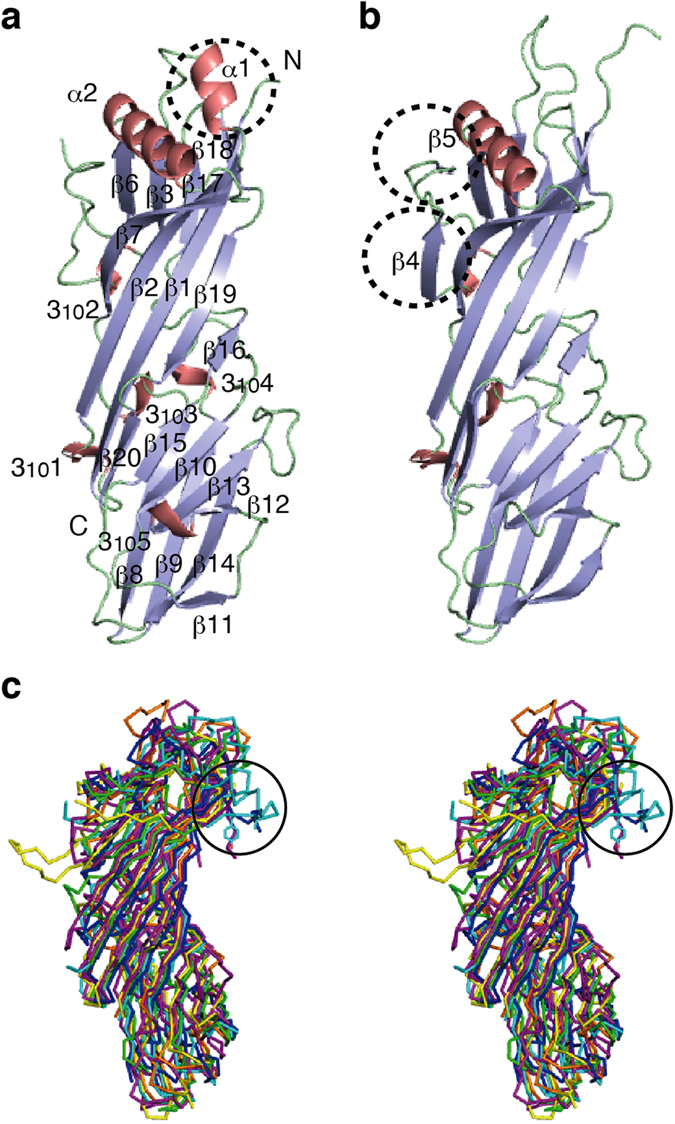
Three-dimensional structures of the μHDs. (**a**,**b**) Ribbon models of the crystal structures of the SGIP1 μHD, in two different space groups. The α- and 3_10_-helices, β-sheets, and coil regions of the μHDs are colored salmon, light blue, and pale green, respectively. (**a**) The selenomethionine (SeMet)-substituted SGIP1 μHD in the *P*42_1_2 space group. N and C indicate the amino and carboxy termini. Secondary structure elements are labeled. The α helix that is missing in the crystal structure of the SGIP1 μHD in the *P*1 space group is indicated by a dashed circle. (**b**) One of the two SGIP1 μHD molecules in the asymmetric unit of the crystal in the *P*1 space group. The β-sheets missing in the crystal structure of the SGIP1 μHD in the *P*42_1_2 space group are indicated by dashed circles and labeled. (**c**) Stereoview of the superimposition of the backbone Cα atoms of the SGIP1 μHD (cyan), the μ2 μHD^23^ (magenta; PDB ID code 1BW8), the μ3 μHD^24^ (green; PDB ID code 4IKN), the μ4 μHD^25^ (yellow; PDB ID code 3L81), the Syp1 μHD^8^ (orange; PDB ID code 3G9H), the bovine COPI δ subunit μHD^26^ (blue; PDB ID code 4O8Q), and the yeast δ-COP subunit μHD^27^ (purple; PDB ID code 5FJZ). The circles indicate the locations of the C-terminal portion of α2 and the following connecting loop and the N-terminal portion of β7 in the SGIP1 μHD, which adopted unique conformations that significantly differed from other known μHD structures. The two conserved residues of SGIP1, Thr667 and Tyr668, which are located in the loop connecting α2 and β7 and at the N-terminus of β7, respectively, and are involved in interactions with Eps15 (see below), are shown as sticks.

To gain further insights into the mechanism of DPF-motif recognition by the μHD, we solved the crystal structures of the SGIP1 μHD in two different space groups (Fig. 3a,b and Table 1). The two structures are similar to each other, and the minor differences in the secondary structure composition are probably due to crystal packing effects (Fig. 3a,b and Supplementary Fig. 1b). The structure of the SGIP1 μHD adopts a topology similar to those of the previously determined μHD structures^8^,^23^,^24^,^25^,^26^,^27^ (Fig. 3c). However, the SGIP1 μHD possesses unique structural features that distinguish it from the other known μHD structures. Among these distinctive features, the most prominent one is the presence of the SGIP1/FCHo1/FCHo2-specific insertion of a relatively long α-helix (α2) between β6 and β7 (Fig. 3a,b and Supplementary Fig. 1b). Due to this insertion, the loop between α2 and β7 and the N-terminal part of β7 also adopt conformations strikingly different from those in other μHDs (Fig. 3c). Since these regions contain critical Eps15 interacting residues (see below), these unique structural features of the SGIP1 μHD are functionally relevant.

### Structures of the SGIP1 μHD in complex with Eps15 fragments

**Figure 4:**
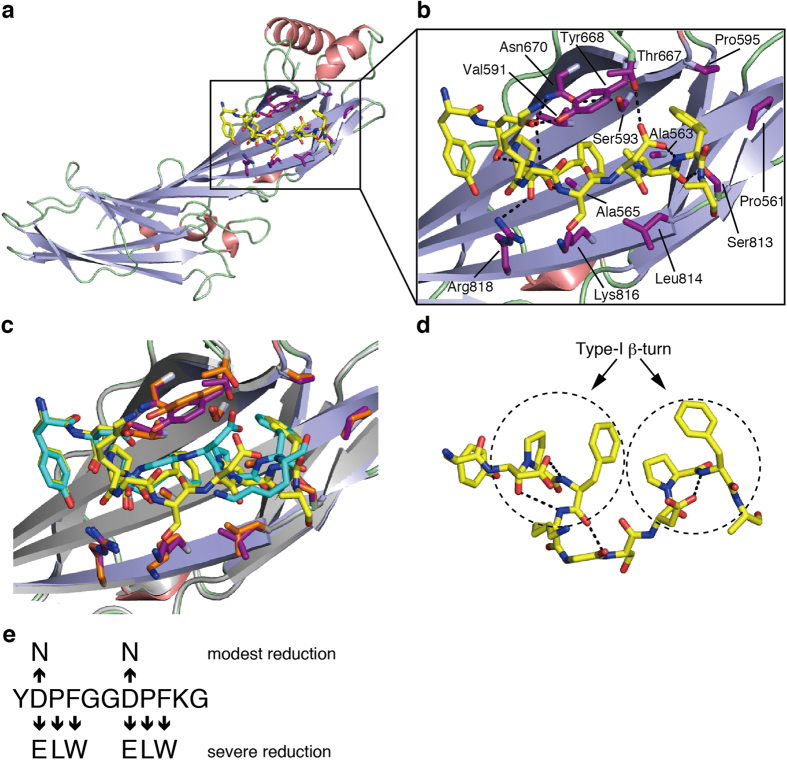
Crystal structures of the SGIP1 μHD in complex with Eps15-derived peptides. (**a**) Ribbon model of the crystal structure of the SGIP1 μHD in complex with Eps15-645–654. The μHD is colored as in Fig. 3a. The SGIP1 residues involved in the recognition of Eps15-645–654 are shown as magenta sticks. Eps15-645–654 is shown as yellow sticks. (**b**) Close-up view of the interaction between the μHD and Eps15-645–654 (amino acid sequence: YDPFKGSDPFA). The molecules are colored as in (**a**). Selected interface residues of the μHD are labeled. Dotted lines indicate intermolecular and intramolecular hydrogen bonding networks involved in Eps15-645–654 recognition by the μHD. (**c**) Superimposition of close-up views of the μHD in complex with Eps15-645–654 and that in complex with Eps15-640–649 (amino acid sequence: YDPFGGDPFKG). The μHD in complex with Eps15-645–654 is colored as in (**a)**. The μHD in the Eps15-640–649 complex is colored gray. Eps15-640–649 and selected interface residues of the μHD in the Eps15-640–649 complex are shown as cyan and orange sticks, respectively. (**d**) The structure of Eps15-645–654 bound to the μHD. Only the Eps15-645–654 molecule is shown, as yellow sticks. Dotted straight lines indicate hydrogen bonds stabilizing the type-I β-turn conformations of the two DPF motifs, indicated by dashed circles. (**e**) A diagram showing the effects of the replacement of each residue of the two DPF motifs with the indicated amino acids on the affinity for the μHD.

**Figure 5:**
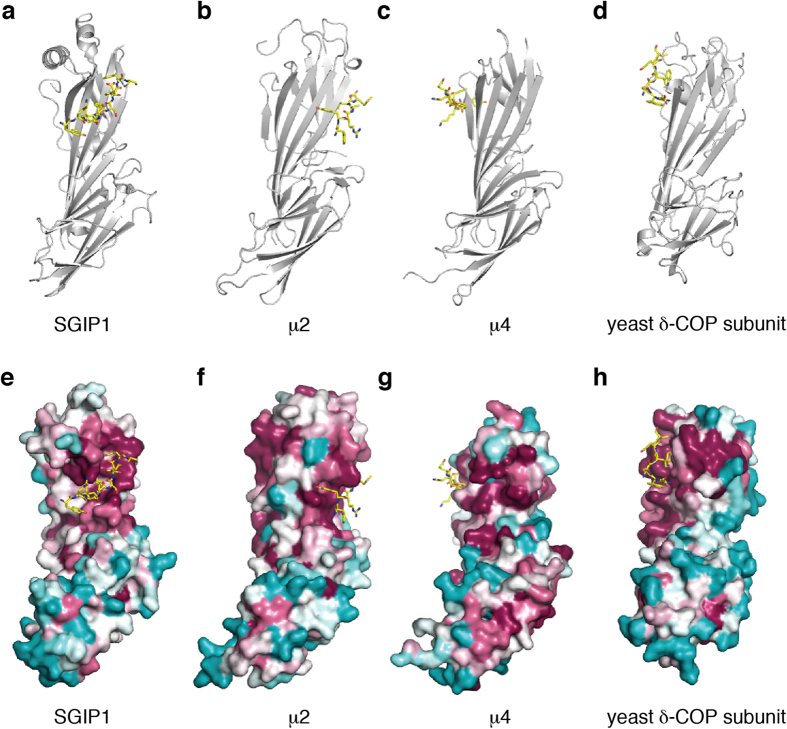
Comparison of the known ligand-binding sites of the μHDs. (**a**–**c**) Complexes of the μHDs of various proteins and their peptide ligands. The μHDs (gray) and peptides (yellow) are shown as ribbon models and sticks, respectively. (**a**) The SGIP1 μHD in complex with Eps15-645–654. (**b**) The μ2 μHD in complex with the peptide FYRALM^23^ (PDB ID code 1BW8). (**c**) The μ4 μHD in complex with the peptide TYKFFEQ^25^ (PDB ID code 3L81). (**d**) The yeast δ-COP subunit μHD in complex with the peptide DWNWEV^27^ (PDB ID code 5FJZ). (**e**–**h**) Conserved surface residues of the μHDs. The bound peptides are shown as in (**a)**–(**d**), respectively. (**e**) The surface of the SGIP1 μHD is colored according to the rate of sequence conservation among the 150 sequences of close homologs^46^, in a gradient from cyan (most variable residues) to white to magenta (most highly conserved residues). (**f**) The surface of the μ2 μHD is colored according to the rate of sequence conservation among the 150 sequences of close homologs, as in (**e**). (**g**) The surface of the μ4 μHD is colored according to the rate of sequence conservation among the 16 sequences of close homologs, as in (**e**). (**h**) The surface of the yeast δ-COP subunit μHD is colored according to the rate of sequence conservation among the 25 sequences of close homologs, as in (**e**).

We next crystallized the complex between the SGIP1 μHD and two short μHD-binding Eps15 fragments containing two DPF motifs derived from the high-affinity μHD-binding site, Eps15-640–649 and Eps15-645–654 (Fig. 1a). We easily obtained crystals of the complexes using the same crystallization conditions as for the SGIP1 μHD alone, and solved their crystal structures (Fig. 4a–c, Supplementary Fig. 4 and Table 1). In the two structures, the two DPF motifs are recognized by a continuous cleft on the μHD (Fig. 4a–c). This binding site is distinct from the known ligand-binding sites of the μHDs of other proteins (Fig. 5). In the two structures, the C-terminal flanking residues and the 2–3 residue linkers between the two DPF motifs form few contacts with the μHD (Fig. 4b,c). The two DPF motifs in the two structures adopt type-I β-turn conformations (Fig. 4d), and each is stabilized by two sequence-specific intramotif interactions. One of the sequence-specific interactions is the hydrogen bond formed between the side chain of Asp and the main-chain amide group of Phe. The other is the hydrophobic interaction between the side chains of Pro and Phe.

In addition to these sequence-specific interactions, a hydrogen bond is formed between the main-chain carbonyl group of Asp and the amide group of the residue three residues down the chain in the N-terminal DPF motif (Fig. 4d). Although this hydrogen bond contributes to the stabilization of the type-I β-turn conformation of the DPF motif, we presume that the two sequence-specific interactions are primarily responsible for the stabilization of the type-I β-turn conformation, as these interactions are observed in both of the DPF motifs. In the two structures, the two DPF motifs bind to the same recognition cleft on the μHD. However, the positions of the C-terminal DPF motif relative to the recognition cleft slightly differ between the two structures, probably due to differences in the lengths and the amino acid sequences of the linker region between the two DPF motifs (Fig. 4c). Nevertheless, the key interactions for DPF motif recognition by the μHD are essentially conserved in the two structures (see below).

### Recognition mode of the two consecutive DPF motifs by the μHD

In our complex structures, the two DPF motifs are intimately packed against each other through hydrophobic interactions between the side chains of Phe in the N-terminal DPF motif and Pro in the C-terminal DPF motif (Fig. 4b–d). This arrangement of the DPF motifs brings the hydrophobic side chains of all of the Pro and Phe residues of the two DPF motifs together on one side, allowing their extensive recognition by the continuous hydrophobic cleft of the μHD (Fig. 4a–d). Two conserved Ala residues, Ala563 and Ala565, of SGIP1 are located at the bottom of this recognition cleft (Fig. 4b). Ala563 contacts the side chains of Phe in the N-terminal DPF motif and Pro in the C-terminal DPF motif, while Ala565 contacts the side chains of Pro and Phe in the N-terminal DPF motif (Fig. 4b). The replacement of either Ala563 or Ala565 with a bulkier and charged Glu abolished the binding to the μHD-binding Eps15 fragment, GST-Eps15-628–654 (Figs 1a and 2 and Supplementary Table 2). The two Asp residues in the two DPF motifs are also specifically recognized by the μHD. First, the two conserved SGIP1 residues, Tyr668 and Asn670, recognize the carboxyl group of Asp in the N-terminal DPF motif (Fig. 4b). Indeed, the replacement of Asn670 with Asp abolished the binding to the μHD-binding Eps15 fragment, Eps15-640–649 (Figs 1a and 2 and Supplementary Table 2). Second, a conserved SGIP1 residue, Thr667, recognizes the side chain of Asp in the C-terminal DPF motif.

A previous report showed that a conserved FCHo2 residue, Lys797, corresponding to the SGIP1 residue Lys816, is critical for the Eps15 interaction^9^. Consistent with this, the replacement of SGIP1 Lys816 with Glu abolished the binding to a μHD-binding Eps15 fragment, Eps15-640–654 (Figs 1a and 2 and Supplementary Table 2). Although Lys816 does not directly contact the Eps15 fragments in our structures, the electrostatic interactions between the side-chain amino group of Lys816 and the main-chain carbonyl groups of Pro and Phe in the N-terminal DPF motif likely stabilize the complex formation (Fig. 4b,c). Arg818 is located close to Lys816 and forms a hydrogen bond with the main-chain carbonyl group of Pro in the N-terminal DPF motif (Fig. 4b). Thus, Arg818 also contributes to the recognition of the main chain in the N-terminal DPF motif. Ser813 also forms a hydrogen bond with the main-chain carbonyl group of Pro in the C-terminal DPF motif (Fig. 4b). Although these residues recognize the main-chain carbonyl groups, they may indirectly contribute to the sequence specificity, because the positions of the main-chain carbonyl groups are restricted by the adoption of the type-I β-turn conformation. Thus, these residues may recognize the type-I β-turn conformation of the fragments.

### Importance of each residue in the two consecutive DPF motifs for μHD binding

We next examined the importance of each residue in the two consecutive DPF motifs for recognition by the μHD. The single replacement of the Asp, Pro, and Phe residues in the two DPF motifs with Glu, Leu, and Trp, respectively, severely reduced the binding to the μHD (Figs 2 and 4e, Supplementary Fig. 3 and Supplementary Table 2). Thus, all six residues of the two consecutive DPF motifs significantly contribute to μHD binding. In contrast, the replacement of Asp in the N-terminal DPF motif with Asn only modestly reduced the binding to the μHD, by ~3.4-fold (Figs 2 and 4e, Supplementary Fig. 3 and Supplementary Table 2). Similarly, the replacement of Asp in the C-terminal DPF motif with Asn also modestly reduced the binding to the μHD, by ~2.4-fold (Figs 2 and 4e, Supplementary Fig. 3 and Supplementary Table 2). The crystal structures of the complexes indicated that the two Asp residues in the DPF motifs play two different roles in μHD binding. One role is to maintain the type-I β-turn conformation required for correctly orienting the Pro and Phe side chains for binding to the hydrophobic recognition cleft of the μHD. The other role is to directly interact with the μHD residues, such as Thr667, Tyr668, and Asn670. The replacement of each Asp residue with Asn did not seem to preclude the formation of the hydrogen bond between the side chain of this Asn and the main-chain amide group of the Phe two residues down the chain, and thus this region could still adopt the type-I β-turn conformation (Fig. 4d). Moreover, the side chains of the μHD residues involved in the recognition of the Asp residues in the DPF motifs seem to be able to accommodate the Asn side chain with rather slight changes in their orientations (Fig. 4b). These facts explain the rather small reduction in the affinity caused by the replacement of Asp with Asn.

### Importance of the linker residues between the two DPF motifs for μHD binding

Although there were few direct contacts between the Eps15 residues outside the DPF motifs and the μHD residues (Fig. 4b), the residues outside the DPF motifs may indirectly affect the strength of their affinity. Indeed, the affinity of a given Eps15 fragment containing two DPF motifs with the μHD significantly varied, depending on the amino acid residues outside the DPF motifs (Figs 1a and 2, Supplementary Fig. 3 and Supplementary Table 2). In our structures of the complexes, the two DPF motifs are intimately packed together and extensively recognized by the μHD (Fig. 4a–d). The conformations of the residues in the linker region are thus likely to be restrained by the direct contacts between the two DPF motifs upon μHD binding. In this case, residues often found in flexible regions, such as Gly, may be required for the Eps15 fragments to adopt a suitable conformation for sufficient binding to the μHD. Indeed, fragments containing two DPF motifs connected by linkers with Gly or other residues with relatively small side chains tended to bind to the μHD more strongly than those without such amino acids, such as Eps15-628–639 and Eps15-797–807 (Figs 1a and 2, Supplementary Fig. 3 and Supplementary Table 2).

### FCHo1 μHD binding to the same high-affinity binding site in Eps15

As the DPF motif-interacting residues in SGIP1 are mostly conserved in the FCHo1/FCHo2 sequences, FCHo1/FCHo2 are expected to interact with Eps15 in a similar manner to that of SGIP1 (Supplementary Fig. 1b). Indeed, the FCHo1 μHD bound strongly to Eps15-618–654, with a *K*_d_ in the nanomolar range, and relatively weakly to Eps15-640–654, with a *K*_d_ of ~5.5 μM (Fig. 2, Supplementary Fig. 3 and Supplementary Table 2). Thus, in principle, the FCHo1 μHD binds to the Eps15-derived fragments containing different numbers of consecutive DPF motifs with affinities comparable to those of the SGIP1 μHD. Note that the ITC data of the FCHo1 μHD binding to Eps15-618–654 were fitted with the single-site model relatively well, unlike the SGIP1 μHD binding to Eps15-618–654 (Supplementary Fig. 3 and Supplementary Table 2). This suggests that the presence of the additional weak binding site in Eps15-618–654 for the SGIP1 μHD is functionally insignificant.

### Non-overlapping high-affinity μHD- and α-adaptin appendage domain-binding sites in Eps15

The α-adaptin appendage domain of the AP-2 complex only weakly binds to a short peptide containing a single DPF motif, with a *K*_d_ of ~100 μM^7^. However, a high-affinity α-adaptin appendage domain-binding site reportedly exists in a region between residues 530 to 791 of Eps15, with a *K*_d_ of ~21 nM^3^. Consistent with this, Eps15 is reportedly constitutively associated with the AP-2 complex, although this association is modified by the perturbation of the interaction between the β2-adaptin of the AP-2 complex and Eps15 on the clathrin assembly^28^,^29^,^30^,^31^,^32^,^33^.

**Figure 6:**
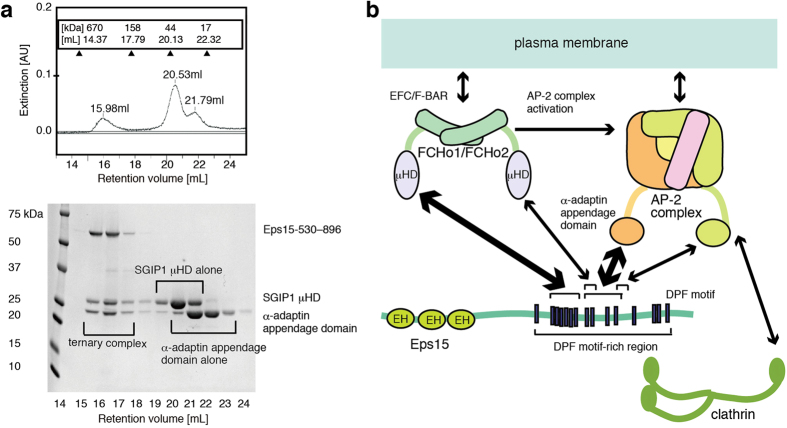
Mutually non-exclusive high-affinity binding of the μHD and the appendage domain to Eps15. (**a**) SDS-PAGE gel pattern of the elution fractions from the gel filtration analysis, showing the equimolar binding of Eps15-530–896, the SGIP1 μHD, and the α-adaptin appendage domain. Eps15-530–896, the SGIP1 μHD, and the appendage domain were mixed in a 1:3:6 molar ratio and analyzed by gel filtration. The Superdex 200 elution profile and the SDS-PAGE analysis of the fractions showed that one peak corresponds to the ternary complex of Eps15-530–896, the SGIP1 μHD, and the appendage domain, and the other two peaks correspond to the SGIP1 μHD alone and the appendage domain alone. (**b**) A model of FCHo1/FCHo2, the AP-2 complex, and Eps15 participating in the clathrin assembly step of CME. The high-affinity interactions between the FCHo1/FCHo2 μHD and Eps15, and between the AP-2 complex and Eps15 are emphasized by the thick double arrows. Only major interactions are shown, for clarity.

To investigate whether Eps15-530–896, the SGIP1 μHD, and the α-adaptin appendage domain form a ternary complex, we analyzed the mixture of Eps15-530–896, the SGIP1 μHD, and the α-adaptin appendage domain by gel filtration, and found that these proteins formed an equimolar complex (Fig. 6a). This result indicates that the high-affinity binding site for the α-adaptin appendage domain in Eps15-530–896 does not overlap with that for the μHD. Moreover, Eps15 fragments outside the high-affinity binding site for the μHD, namely Eps15-661–790 (Fig. 1a) and Eps15-661–720 (spanning residues 661 to 720), strongly bound to the α-adaptin appendage domain, with *K*_d_ values in the nanomolar range (Fig. 2, Supplementary Fig. 3 and Supplementary Table 2). Thus, the region spanning residues 661 to 720 contains the high-affinity binding site for the α-adaptin appendage domain. This region is slightly shorter than the region in Eps15 (residues 667–739) that was previously identified as the major α-adaptin appendage domain-binding site by pull-down assays^6^. Altogether, these data suggest that SGIP1/FCHo1/FCHo2, Eps15, and the AP-2 complex form a tight complex. This conclusion is consistent with the coincident appearance of Eps15 and FCHo1/FCHo2 as puncta at the plasma membrane^9^. In contrast, these proteins are not always co-localized with the AP-2 complex at the plasma membrane in the process preceding clathrin assembly^9^, indicating the existence of a regulatory mechanism that modifies the interaction between the AP-2 complex and Eps15 in this process.

## Discussion

For most higher eukaryotes, Eps15 recruitment by FCHo1/FCHo2 plays a critical role in the accumulation of the AP-2 complex at the plasma membrane, which culminates in clathrin assembly, although for some species, the degrees of its contribution to clathrin assembly are less significant^9^,^17^,^22^. We identified the high-affinity binding site in Eps15 for the SGIP1/FCHo1 μHDs, which is composed of six consecutive DPF motifs connected by 2–3 residue linkers. We also determined the crystal structures of the complexes between the SGIP1 μHD and the Eps15 fragments containing two consecutive DPF motifs, which are the minimal μHD-binding unit.

This recognition mode clearly explains how two endocytic proteins, Dab2 and Eps15R, bind to the FCHo2 μHD^9^,^11^, as these are the only proteins other than Eps15 encoded in the human genome with at least one set of two consecutive DPF motifs connected by 2–3 residue linkers, according to database searches. As Dab2 contains only one set of two consecutive DPF motifs, the recognition of the two consecutive DPF motifs is a more widely distributed function of the SGIP1/FCHo1/FCHo2 μHD than the high affinity recognition of the six consecutive DPF motifs, which is only applicable to Eps15 and Eps15R. Eps15, Eps15R, and Dab2 are all components of CCPs^9^,^11^,^34^. Our results suggest that in cells expressing all three of these proteins, they are likely to compete with each other for binding to the SGIP1/FCHo1/FCHo2 μHD. Thus, either Eps15, Eps15R, or Dab2 predominantly binds to the SGIP1/FCHo1/FCHo2 μHD and plays a major role in clathrin assembly dependent on various conditions, such as their affinities for the μHD, the cellular expression levels, and the association with membrane-localized specific cargo. The other two are probably minor components of CCPs or negatively regulate CME in a competitive manner. CCPs with distinct compositions of Eps15, Eps15R, and Dab2 should display distinguishing properties suitable for the internalization of specific cargo. Dab2 reportedly arrives at CCPs later than FCHo1/FCHo2 and Eps15 and after clathrin^9^. Thus, Eps15 and Dab2 may function in a sequential manner in clathrin assembly in certain cell types, where the binding partners of the μHDs of SGIP1/FCHo1/FCHo2 may be switched from Eps15 to Dab2 during the maturation of the CCPs.

Similarly, SGIP1/SGIP1α, FCHo1, and FCHo2 are likely to compete with each other for binding to the consecutive DPF motifs in Eps15/Eps15R/Dab2, when they are expressed in the same cell. Depending on various conditions, such as their expression levels, one of them predominantly binds to Eps15/Eps15R/Dab2 and plays a major role in clathrin assembly. The other two proteins are probably minor components of CCPs or negatively regulate CME in a competitive manner. This latter prediction is consistent with the fact that the overexpression of SGIP1α reduced the CME of two types of transport cargo, transferrin and EGF^19^, probably due to the competitive inhibition of the FCHo1/FCHo2 function by the overexpressed SGIP1α. In contrast, the knockdown of SGIP1α reduced the transferrin endocytosis, but not the EGF endocytosis. This suggests that CCPs containing SGIP1α as a component are more effective in the CME of transferrin. SGIP1 reportedly arrives at the site of CME later than FCHo1/FCHo2 and after the AP-2 complex^9^. Thus, it is also possible that SGIP1 plays a role in a later step of clathrin assembly, distinct from those of FCHo1/FCHo2, in which the switching of the binding partners of Eps15/Eps15R/Dab2 from FCHo1/FCHo2 to SGIP1 may be involved.

While FCHo1/FCHo2 are ubiquitous CCP nucleators^9^, SGIP1/SGIP1α are predominantly expressed in the brain^18^,^19^. Thus, the predicted functions of SGIP1/SGIP1α discussed above may operate only in the brain. SGIP1 is implicated in energy homeostasis and obesity in mice, rats, and humans^18^,^35^. These findings suggest the hypothetical role of SGIP1/SGIP1α in the CME of specific cargo in the brain, which ultimately controls the feeding behavior of mammals. As the selective reduction of the expression level of SGIP1 resulted in the inhibition of food intake and the reduction of body weight in rat models of obesity and diabetes^18^, SGIP1 seems to be a potential therapeutic target for obesity- and diabetes-related symptoms. Interestingly, the sizes of CCPs in rat and mouse brains are significantly smaller than those generally observed in mouse or human epithelial cells^1^. This unique property of the brain vesicles may exist because large extracellular molecules do not need to be internalized in synaptic vesicle recycling^1^. Thus, the functions of SGIP1/SGIP1α discussed above may play a role in controlling the size of the brain vesicles. As SGIP1 orthologs are present in a wide range of vertebrates, such as zebrafish, frog, and chicken, the SGIP1 orthologs within these species may also play physiological roles similar to those within mouse, rat, and human.

An inspection of the literature and our results revealed no clear difference between the μHDs of SGIP1/SGIP1α, FCHo1, and FCHo2, in terms of their properties to recognize the consecutive DPF motifs in Eps15/Eps15R/Dab2 (Supplementary Table 3). However, a more detailed characterization of these μHDs may identify the SGIP1 μHD-specific characteristics involved in DPF motif recognition. This would enable the development of SGIP1-specific low molecular weight inhibitors, which could serve as research tools to investigate the functions of SGIP1/SGIP1α in more detail, as well as therapeutic agents. However, it seems more likely that the major differences in the endocytic properties between SGIP1/SGIP1α and FCHo1/FCHo2 reside in the regions outside the μHD or in the abilities of their μHDs to bind to distinct endocytic binding partners, such as Hrb and CALM, which lack DPF motifs and interact only with the FCHo1 μHD, but not with the FCHo2 μHD^22^. Further investigations are required to understand the complex interplay of SGIP1/SGIP1α/FCHo1/FCHo2 and Eps15/Eps15R/Dab2 in the regulation of the CME of different kinds of cargo in various cell types.

Our structures revealed that the SGIP1 μHD possesses a binding cleft for two consecutive DPF motifs. This is the first example of the simultaneous binding of two DPF motifs to a single domain for tighter binding. A large portion of the short repeated motifs found in endocytic proteins are scattered throughout a single polypeptide and have multiple different binding partners, as in the case of the Eps15 DPF motifs^3^,^11^. The simultaneous recognition of two short motifs is certainly beneficial for a protein to select correct binding sites, in a region containing many short motifs. In contrast, the α-adaptin appendage domain uses two remote ligand-binding sites on the domain for tight binding to Eps15^3^,^36^, where one of the two sites presumably binds to one of the DPF motifs in Eps15 and the other site binds to a different, unknown sequence. Thus, this study clearly showed that the fifteen DPF motifs in Eps15 are not functionally equivalent, and are recognized differentially by the SGIP1/FCHo1/FCHo2 μHDs and the α-adaptin appendage domain. This recognition mode is also consistent with the expected *in vivo* function of FCHo1/FCHo2, Eps15, and the AP-2 complex in clathrin assembly, which requires the simultaneous binding of the FCHo1/FCHo2 μHD and the AP-2 complex to Eps15^9^,^10^ (Fig. 6b).

A previous report showed that the second EH domain of Eps15 contains separate binding sites for two NPF motifs of stonin2, which are thirteen residues apart, and the binding of both NPF motifs was required for a tighter interaction^37^. Thus, the binding of two DPF motifs to the μHD is the second structurally verified example of the binding of two DPF-related motifs to a single domain. Although there is some similarity between these systems, there is also a major difference. In the case of the recognition of the two DPF motifs by the μHD, the DPF motifs interact not only with the μHD but also with each other, to maintain conformations favorable for μHD binding. This intermotif interaction seems to be critical for binding to the hydrophobic recognition cleft on the μHD. Thus, the recognition mode identified in this study is novel.

The two DPF motifs bound to the μHD adopt the type-I β-turn conformations often found in the structures of DPF-related motifs, such as those of the DPF, NPF and DPW motifs bound to their binding partners^37^,^38^,^39^. In principle, the two key intramotif interactions described in this study, which stabilize the type-I β-turn conformation, could also be maintained in the NPF and DPW motifs, due to the common properties of their side chains. Thus, these motifs may possess a propensity to adopt the type-I β-turn conformation in solution, due to the properties encoded in their sequences. This suggests that the binding partners of these motifs recognize them by conformational selection, rather than an induced fit mechanism.

In our structures of the complexes, the two DPF motifs intimately interacted with each other to form a unit suitable for μHD binding. In solution, the μHD likely recognizes this ordered structure, stabilized by the intramotif and intermotif interactions that this unit tends to adopt, regardless of whether it is stable or transiently formed. A well-known example of a locally ordered structure, formed in a largely unstructured region, that acts as binding sites for other proteins is the polyproline II helix often found in proline-rich sequences. The polyproline II helix is recognized by numerous specific binding partners, such as the SH3 domain^40^,^41^. Thus, this study highlights the importance of understanding the functions of short sequence motifs based on their locally ordered structures in solution.

The mechanism underlying the enhancement of the affinity for the μHD by the increase in the number of consecutive DPF motifs is currently unclear. We propose several possible mechanisms, as working hypotheses. The first possible mechanism is that there are additional, as yet unidentified, DPF motif-binding sites in the μHD. The second mechanism is that the presence of more binding sites for the same binding cleft in the μHD facilitates the rebinding of the μHD to the fragments with more consecutive DPF motifs, leading to affinity enhancement. The third mechanism is that the increase in the negative charges, due to the Asp residue side chains in the DPF motifs, enhances the affinity by electrostatic interactions with the μHD. The fourth mechanism is that the neighboring DPF motifs in the fragment mutually restrain their conformations, resulting in a reduction in the dynamics and thus the conformational entropy of the fragment in the unbound state. This effect reduces the entropic penalty upon binding to the μHD, and thus contributes to the affinity enhancement. We are currently testing these possible mechanisms by a variety of crystallographic, biochemical, and biophysical techniques. The results will be published elsewhere.

## Methods

### Protein and peptide preparations

The μHD of human SGIP1 (reference sequence NM_032291) was expressed as a glutathione S-transferase (GST)-fusion protein, using the pGEX-6P-1 vector (GE Healthcare) and either *Escherichia coli* BL21-Gold (DE3) (Agilent Technologies) or Rosetta2 (Novagen) as the host strain. The cells expressing the GST-fusion protein were grown at 37 °C, induced at an A_600_ of 0.8 with 200 μM isopropyl-β-D-thiogalactopyranoside (IPTG), and incubated overnight at 20 °C. The cells were harvested, resuspended in 50 mM Tris-HCl buffer (pH 8.0) containing 50 mM NaCl, 5 mM 1,4-dithiothreithol (DTT), 2 mM MgCl_2_, 10% glycerol (buffer A), one tablet of cOmplete EDTA-free protease inhibitor (Roche) per 50 mL solution, and 5 μL Benzonase Nuclease (Merck Millipore), and homogenized by sonication. After centrifugation, the supernatant fraction was loaded onto a Glutathione Sepharose 4B (GE Healthcare) column equilibrated with buffer A. The column was sequentially washed with 50 mM Tris-HCl buffer (pH 8.0) containing 400 mM NaCl, 5 mM DTT, and 10% glycerol; 50 mM Tris-HCl buffer (pH 8.0) containing 100 mM KCl, 10 mM MgCl_2_, 5 mM DTT, 0.25 mM ATP, and 5% glycerol; and 50 mM Tris-HCl buffer (pH 6.8) containing 150 mM NaCl, 1 mM EDTA (pH 8.0), and 1 mM DTT (buffer B). After 3C protease cleavage on the column overnight, the protein was eluted with buffer B. The protein was further purified by Superdex 200 gel filtration in 50 mM Tris-HCl buffer (pH 8.0), containing 50 mM NaCl, 5 mM DTT, and 5% glycerol (buffer C). The protein ran as a single band on an SDS-PAGE gel, and was typically concentrated to 7.0 mg/mL with a Vivaspin Turbo 15 centrifugal concentrator (Sartorius). The SeMet-substituted SGIP1 μHD was expressed using the host *E. coli* strain B834(DE3) (Novagen), in selenomethionine core medium (Wako) supplemented with 50 mg/L L-selenomethionine (Nacalai Tesque). The expressed SeMet-substituted SGIP1 μHD was purified in the same manner as for the native SGIP1 μHD, and was typically concentrated to 7.0 mg/mL. The expression vectors encoding the GST-fused SGIP1 μHD mutants were created by introducing the mutations into the vector encoding the GST-fused SGIP1 μHD, according to the QuikChange protocol. The SGIP1 μHD mutants were expressed and purified in the same manner as for the SGIP1 μHD. The C-terminal fragment of human Eps15 (reference sequence NM_001981), Eps15-530–896, was expressed as an N-terminally GST-fused and C-terminally His-tagged protein, and was harvested, sonicated, and centrifuged in the same manner as for the SGIP1 μHD. After centrifugation, the supernatant fraction was dialyzed overnight against 50 mM Tris-HCl buffer (pH 8.0), containing 50 mM NaCl and 10% glycerol (buffer D), and was loaded onto a Ni Sepharose High Performance (GE Healthcare) column equilibrated with buffer D. The column was washed with 20 mM Tris-HCl buffer (pH 8.0), containing 500 mM NaCl and 10 mM imidazole. The protein was eluted with 20 mM Tris-HCl buffer (pH 8.0), containing 500 mM NaCl and 500 mM imidazole. The protein was then dialyzed overnight against 50 mM Tris-HCl buffer (pH 8.0), containing 50 mM NaCl, 10 mM KCl, and 5 mM MgCl_2_. Subsequently, the protein was digested with thrombin for His-tag removal. The protein was then loaded onto a Glutathione Sepharose 4B column equilibrated with buffer A. The column was then washed with buffer A. For the rest of the purification steps, the protein was treated in the same manner as for the SGIP1 μHD. The protein was typically concentrated to 7.0 mg/mL. The other Eps15 fragments longer than thirty amino acids, the μHD of human FCHo1 (reference sequence NP_001154830) spanning residues 623 to 889, and the appendage domain of human α-adaptin (reference sequence NP_570603) spanning residues 711 to 955 were expressed and purified in the same manner as for the SGIP1 μHD. Eps15 fragments with the GST-tag were expressed, and were treated in the same manner as for the SGIP1 μHD up to the Glutathione Sepharose 4B column loading step and the column washing step with 50 mM Tris-HCl buffer (pH 8.0), containing 100 mM KCl, 10 mM MgCl_2_, 5 mM DTT, 0.25 mM ATP, and 5% glycerol. The column was then washed with buffer C. The proteins were eluted by buffer C supplemented with 20 mM reduced glutathione. The proteins were further purified by Superdex 200 gel filtration, in the same manner as for the SGIP1 μHD. The rest of the purification steps were performed in the same manner as for the SGIP1 μHD. Peptides shorter than thirty-one residues were custom synthesized with an N-terminal acetyl group and a C-terminal amide group, by Eurofins Genomics (Tokyo, Japan).

### Analytical gel filtration

Analytical gel filtration was performed on a Superdex 200 column, in 50 mM Tris-HCl buffer (pH 8.0), containing 50 mM NaCl, 5 mM DTT, and 5% glycerol.

### ITC measurements

For the majority of the ITC experiments, the SGIP1 μHD, its mutants, or the FCHo1 μHD, in 50 mM Tris-HCl buffer (pH 8.0) containing 50 mM NaCl, was loaded in the sample cell, in a volume of 1.42 ml, and titrated with various Eps15-derived fragments or peptides in the same buffer, with an initial 5 μl injection and subsequent 10 μl injections, for a total of 25 injections. The titrations were performed while the samples were stirred at 310 r.p.m. at 25˚C. Each injection was followed by an interval of 4 min, to allow baseline stabilization. For the other ITC experiments indicated in Supplementary Table 2, the Eps15-derived fragments or peptides in the sample cell were titrated with the SGIP1 or FCHo1 μHD. All ITC experiments were performed on a VP-ITC microcalorimeter (MicroCal, LLC) and repeated twice, and the data were processed with the program NITPIC^42^. The two data sets from each ITC experiment were analyzed by global weighted least-squares fitting with the program SEDPHAT^43^. Figures for the ITC data were created with the program GUSSI (http://biophysics.swmed.edu/MBR/software.html). To obtain the values of the traditional stoichiometric parameter N in our ITC data analyses (Supplementary Table 2), we used the values of the parameters of the incompetent fractions of proteins and ligands^43^. These parameters are defined as the fractions of proteins and ligands that do not participate in binding due to various reasons, such as partial inactivation, and are used in SEDPHAT as floating parameters for curve fitting. The values of the incompetent fractions are between 0 and 1, whereas the other parameter affecting the traditional parameter N, the number of sites, was fixed as 1 and 2, for the single- and two-site models, respectively. Thus, in the two-site model, the values of N for the first and second sites are identical in our ITC data analyses (Supplementary Table 2).

### Crystallization, data collection, and structure determination

The protein solutions of the SGIP1 μHD alone and in complex with Eps15-derived peptides were prepared in buffer C. For the crystallization of the complexes, the μHD and the Eps15-derived peptides were mixed to yield a protein-peptide mixture containing final concentrations of 3.5 mg/mL μHD and 500 μM peptides. All of the crystals used in this study were grown at 20 °C using the hanging-drop vapor-diffusion method, by mixing the protein or protein-peptide mixture in a 1:1 volume ratio with a solution containing 10–18% (w/v) PEG3, 350 (Hampton Research), 150 mM zinc acetate, 100 mM sodium acetate (pH 5.1), and 100 mM sodium iodide. The crystals were flash-cooled in liquid nitrogen, using 10% glycerol as a cryoprotectant. Data sets were collected at the RIKEN Structural Genomics Beamlines I (BL26B1) and II (BL26B2), the SPring-8 beamlines BL44XU and BL32XU (Hyogo, Japan), and the Photon Factory beamline BL-1A (Tsukuba, Japan), and were processed with HKL2000^44^. The structure of the SeMet-substituted SGIP1 μHD in the *P*42_1_2 space group was solved by the SAD method, using the program PHENIX^45^. The native SGIP1 μHD structure in the *P*1 space group and those in complex with Eps15-derived peptides in the *P*42_1_2 space group were solved by the molecular replacement method, using the structure of the SeMet-substituted SGIP1 μHD as a search model with PHENIX. Refinement and model building were performed with PHENIX. Figures were created with the program PyMol ( http://www.pymol.org).

## Additional Information

**Accession codes:** The coordinates and structure factors have been deposited in the Protein Data Bank, with the ID codes 5AWR, 5AWS, 5AWT, and 5AWU, for the SGIP1 μHD in the *P*4212 space group, the SGIP1 μHD in the *P*1 space group, and the SGIP1 μHD in complex with Eps15-640–649, and with Eps15-645–654, respectively.

**How to cite this article**: Shimada, A. *et al.* Structural basis for the recognition of two consecutive mutually interacting DPF motifs by the SGIP1 µ homology domain. *Sci. Rep.* **6**, 19565; doi: 10.1038/srep19565 (2016).

## Supplementary Material

Supplementary Information

## Figures and Tables

**Table 1:** Data collection and refinement statistics.

	SGIP1 μHD(SeMet)	SGIP1 μHD(Native)	Eps15-640–649complex	Eps15-645–654complex
Data collection				
Space group	*P*42_1_2	*P*1	*P*42_1_2	*P*42_1_2
Cell dimensions				
*a*, *b*, *c* (Å)	109.8, 109.8, 79.5	37.6, 53.6, 75.2	107.7, 107.7, 80.0	109.6, 109.6, 80.1
α, β, γ(°)	90.0, 90.0, 90.0	101.9, 86.9, 95.6	90.0, 90.0, 90.0	90.0, 90.0, 90.0
Resolution (Å)	50.0–2.5 (2.54–2.50)	50.0–2.0 (2.03–2.00)	50.0–2.7 (2.75–2.70)	50.0–2.7 (2.75–2.70)
*R*_merge_	0.127 (>1)	0.083 (0.472)	0.104 (>1)	0.238 (>1)
*I*/σ*I*	25.7 (2.5)	15.0 (2.0)	26.8 (2.1)	9.8 (2.2)
Completeness (%)	99.9 (99.9)	89.1 (53.4)	100.0 (99.7)	99.8 (100.0)
Redundancy	32.4 (26.8)	3.9 (3.2)	14.1 (11.9)	12.0 (10.9)
Refinement				
Resolution (Å)	50.0–2.5 (2.54–2.50)	50.0–2.0 (2.03–2.00)	50.0–2.7 (2.75–2.70)	50.0–2.7 (2.75–2.70)
No. reflections	17,337	34,307	13,428	14,137
*R*_work_/*R*_free_	0.199/0.247	0.199/0.241	0.188/0.234	0.207/0.245
No. atoms				
Protein	2,066	4,175	2,138	2,137
Ion	4	12	6	4
Water	38	188	35	8
*B*-factors				
Protein	73.5	57.0	65.8	94.2
Ion	103.7	101.3	122.0	138.1
Water	59.3	50.3	51.4	63.7
R.m.s. deviations				
Bond lengths (Å)	0.008	0.008	0.022	0.015
Bond angles (°)	1.14	1.39	1.45	1.20

Each structure was determined from a single crystal. Values in parentheses are for highest-resolution shell.
