# Supplementary material for: Structural basis for the recognition of two consecutive mutually interacting DPF motifs by the SGIP1 μ homology domain
Source: Sci Rep. 2016 Jan 29;6:19565. doi: 10.1038/srep19565 (PMC4731787; doi:10.1038/srep19565)
Supplement: Supplementary Information [file srep19565-s1.pdf]

Structural basis for the recognition of two consecutive mutually interacting DPF motifs by the SGIP1 μ homology domain

Atsushi Shimada[1,2,]*, Atsuko Yamaguchi[1], and Daisuke Kohda[1]

[1]Division of Structural Biology, Medical Institute of Bioregulation, Kyushu University, 3-1-1 Maidashi, Higashi-ku, Fukuoka 812-8582, Japan. [2]RIKEN Structural Biology Laboratory, 1-7-22 Suehiro-cho, Tsurumi, Yokohama 230-0045, Japan.

*Corresponding author. Division of Structural Biology, Medical Institute of Bioregulation, Kyushu University, 3-1-1 Maidashi, Higashi-ku, Fukuoka 812-8582, Japan.

Tel: +81 92 642 6969; Fax: +81 92 642 6833; E-mail: ashimada@bioreg.kyushu-u.ac.jp

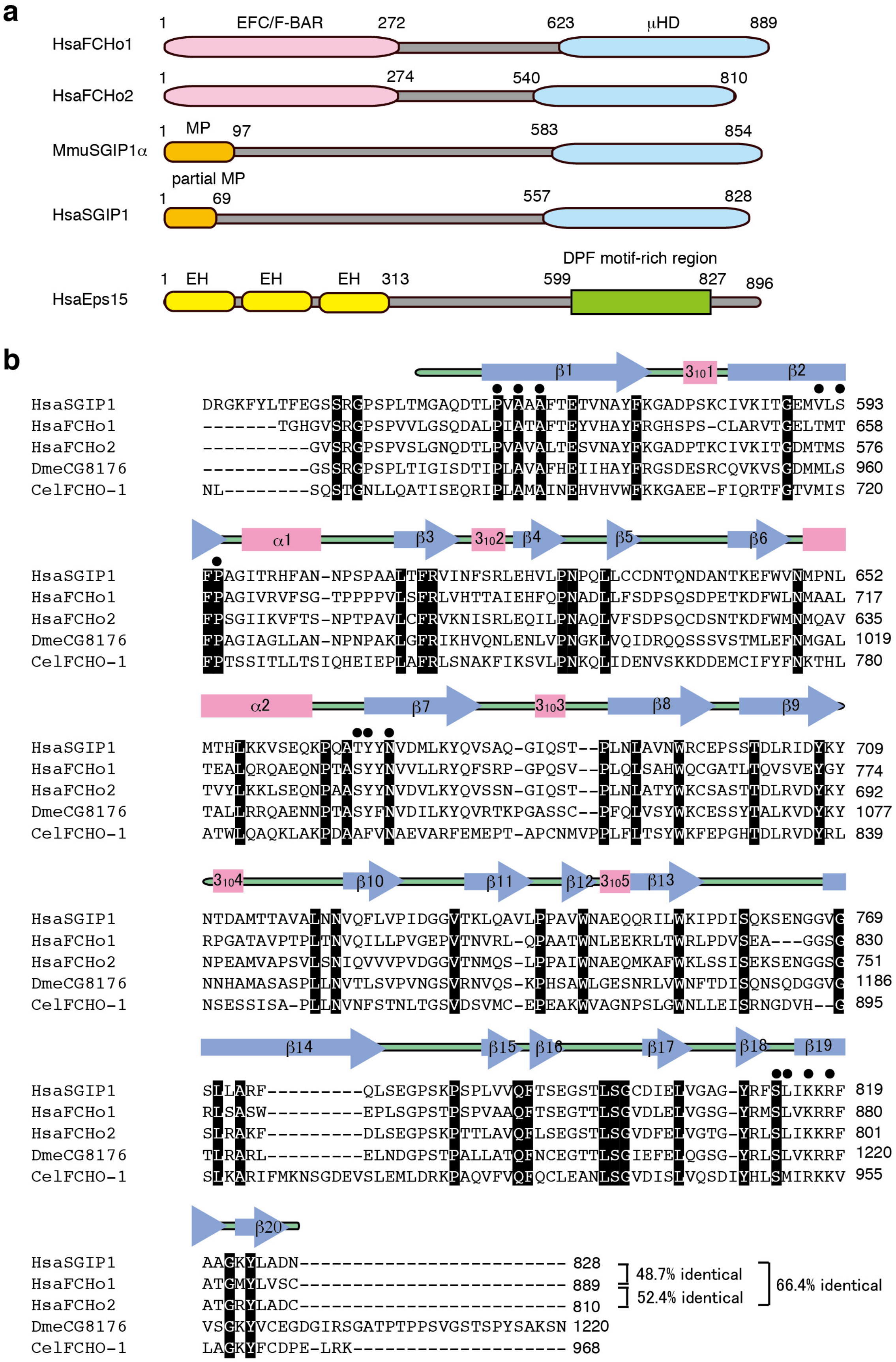
a
HsaFCHo1
1
EFC/F-BAR
272
623
μHD
889
HsaFCHo2
1
274
540
810
MmuSGIP1α
1
MP
97
583
854
partial MP
HsaSGIP1
1
69
557
828
DPF motif-rich region
HsaEps15
1
EH
EH
EH
313
599
827
896
b
β1 3101 β2
HsaSGIP1 DRGKFYLTFEGSSRGPSPLTMGAQDTLPVAAAFTETVNAYFKGADPSKCIVKITGEMVLS 593
HsaFCHo1 -------TGHGVSRGPSPVVLGSQDALPIATAFTEYVHAYFRGHSPS-CLARVTGELTMT 658
HsaFCHo2 ----------GVSRGPSPVSLGNQDTLPVAVALTESVNAYFKGADPTKCIVKITGDMTMS 576
DmeCG8176 ----------GSSRGPSPLTIGISDTIPLAVAFHEIIHAYFRGSDESRCQVKVSGDMMLS 960
CelFCHO-1 NL--------SQSTGNLLQATISEQRIPLAMAINEHVHVWFKKGAEE-FIQRTFGTVMIS 720
α1 β3 3102 β4 β5 β6
HsaSGIP1 FPAGITRHFAN-NPSPAALTFRVINFSRLEHVLPNPQLLCCDNTQNDANTKEFWVNMPNL 652
HsaFCHo1 FPAGIVRVFSG-TPPPPVLSFRLVHTTAIEHFQPNADLLFSDPSQSDPETKDFWLNMAAL 717
HsaFCHo2 FPSGIIKVFTS-NPTPAVLCFRVKNISRLEQILPNAQLVFSDPSQCDSNTKDFWMNMQAV 635
DmeCG8176 FPAGIAGLLAN-NPNPAKLGFRIKHVQNLENLVPNGKLVQIDRQQSSSVSTMLEFNMGAL 1019
CelFCHO-1 FPTSSITLLTSIQHEIEPLAFRLSNAKFIKSVLPNKQLIDENVSKKDDEMCIFYFNKTHL 780
α2 β7 3103 β8 β9
HsaSGIP1 MTHLKKVSEQKPQATYYNVDMLKYQVSAQ-GIQST--PLNLAVNWRCEPSSTDLRIDYKY 709
HsaFCHo1 TEALQRQAEQNPTASYYNVVLLRYQFSRP-GPQSV--PLQLSAHWQCGATLTQVSVEYGY 774
HsaFCHo2 TVYLKKLSEQNPAASYYNVDVLKYQVSSN-GIQST--PLNLATYWKCSASTTDLRVDYKY 692
DmeCG8176 TALLRRQAENNPTASYFNVDILKYQVRTKPGASSC--PFQLVSYWKCESSYTALKVDYKY 1077
CelFCHO-1 ATWLQAQKLAKPDAAFVNAEVARFEMEPT-APCNMVPPLFLTSYWKFEPGHTDLRVDYRL 839
3104 β10 β11 β12 3105 β13
HsaSGIP1 NTDAMTTAVALNNVQFLVPIDGGVTKLQAVLPPAVWNAEQQRILWKIPDISQKSENGGVG 769
HsaFCHo1 RPGATAVPTPLTNVQILLPVGEPVTNVRL-QPAATWNLEEKRLTWRLPDVSEA---GGSG 830
HsaFCHo2 NPEAMVAPSVLSNIQVVVPVDGGVTNMQS-LPPAIWNAEQMKAFWKLSSISEKSENGGSG 751
DmeCG8176 NNHAMASASPLLNVTLSVPVNGSVRNVQS-KPHSAWLGESNRLVWNFTDISQNSQDGGVG 1186
CelFCHO-1 NSESSISA-PLLNVNFSTNLTGSVDSVMC-EPEAKWVAGNPSLGWNLLEISRNGDVH--G 895
β14 β15 β16 β17 β18 β19
HsaSGIP1 SLLARF---------QLSEGPSKPSPLVVQFTSEGSTLSGCDIELVGAG-YRFSLIKKRF 819
HsaFCHo1 RLSASW---------EPLSGPSTPSPVAAQFTSEGTTLSGVDLELVGSG-YRMSLVKRRF 880
HsaFCHo2 SLRAKF---------DLSEGPSKPTTLAVQFLSEGSTLSGVDFELVGTG-YRLSLIKKRF 801
DmeCG8176 TLRARL---------ELNDGPSTPALLATQFNCEGTTLSGIEFELQGSG-YRLSLVKRRF 1220
CelFCHO-1 SLKARIFMKNSGDEVSLEMLDRKPAQVFVQFQCLEANLSGVDISLVQSDIYHLSMIRKKV 955
β20
HsaSGIP1 AAGKYLADN------------------------- 828
HsaFCHo1 ATGMYLVSC------------------------- 889
HsaFCHo2 ATGRYLADC------------------------- 810
DmeCG8176 VSGKYVCEGDGIRSGATPTPPSVGSTSPYSAKSN 1220
CelFCHO-1 LAGKYFCDPE-LRK-------------------- 968
48.7% identical
52.4% identical
66.4% identical

Supplementary Figure 1 Sequence alignment of the μHDs of SGIP1/FCHo1/FCHo2 homologs. (**a**) Domain structures of human FCHo1, human FCHo2, mouse SGIP1α, human SGIP1, and human Eps15. Domain boundaries are indicated. (**b**) Sequence alignment of the *μ*HDs of SGIP1, FCHo1, FCHo2, and their *Drosophila melanogaster* and *Caenorhabditis elegans* homologs. Conserved residues are highlighted. Residues involved in the interactions with the Eps15-derived fragments in our crystal structures are indicated by black dots above the alignment. Secondary structure elements of the SGIP1 *μ*HD, determined from the crystal structure, are shown above the alignment and labeled. The α- and 3_{10}-helices, β-sheets, and coil regions of the μHDs are colored salmon, light blue, and pale green, respectively. The secondary structure elements were determined based on the crystal structures of the SGIP1 *μ*HD in the $P42_12$ space group and that in the $P1$ space group.

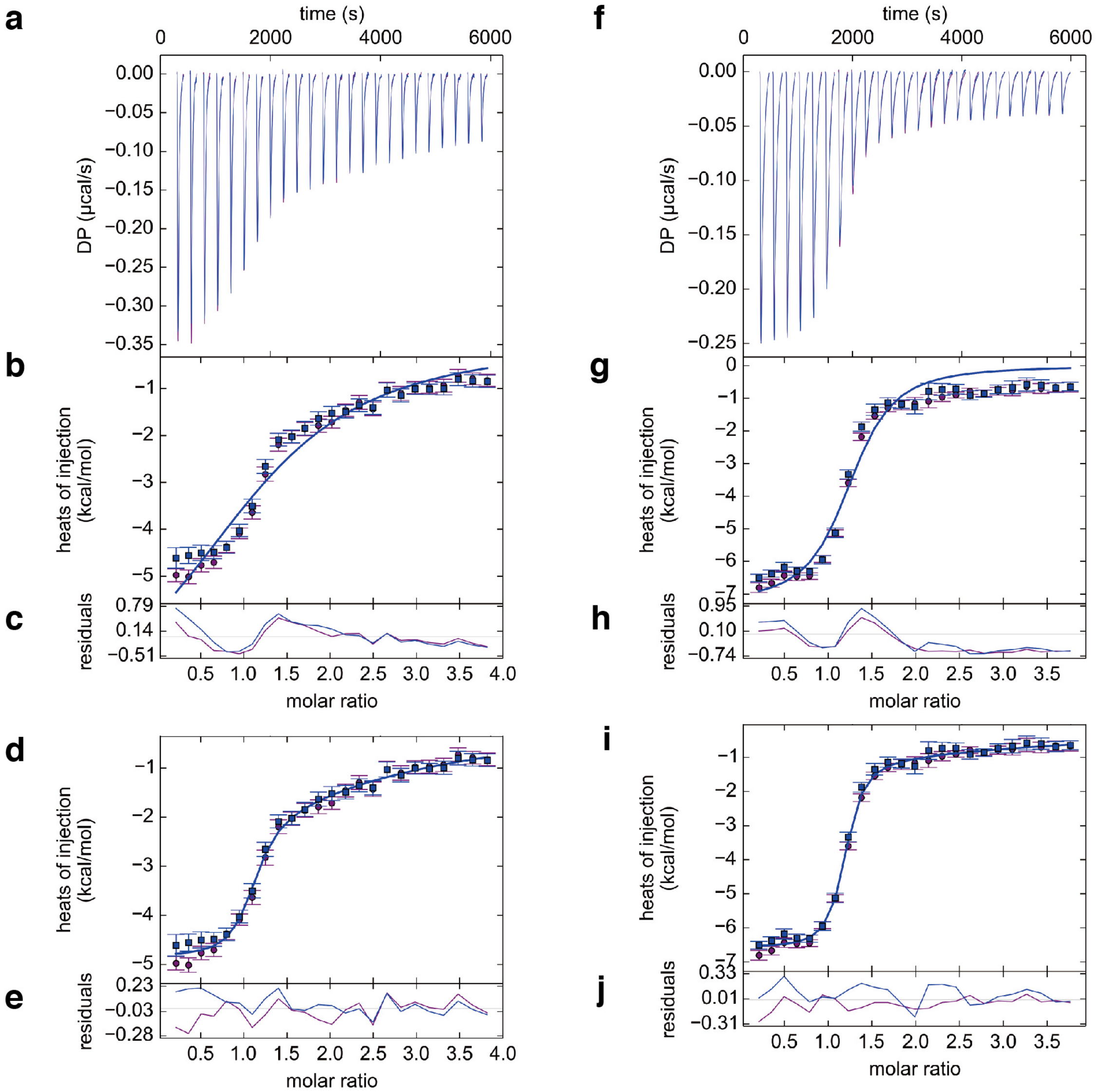
a
time (s)
0
2000
4000
6000
0.00
−0.05
−0.10
−0.15
−0.20
−0.25
−0.30
−0.35
DP (µcal/s)
b
heats of injection (kcal/mol)
−1
−2
−3
−4
−5
c
residuals
0.79
0.14
−0.51
0.5 1.0 1.5 2.0 2.5 3.0 3.5 4.0
molar ratio
d
heats of injection (kcal/mol)
−1
−2
−3
−4
−5
e
residuals
0.23
−0.03
−0.28
0.5 1.0 1.5 2.0 2.5 3.0 3.5 4.0
molar ratio
f
time (s)
0
2000
4000
6000
0.00
−0.05
−0.10
−0.15
−0.20
−0.25
DP (µcal/s)
g
heats of injection (kcal/mol)
0
−1
−2
−3
−4
−5
−6
−7
h
residuals
0.95
0.10
−0.74
0.5 1.0 1.5 2.0 2.5 3.0 3.5
molar ratio
i
heats of injection (kcal/mol)
−1
−2
−3
−4
−5
−6
−7
j
residuals
0.33
0.01
−0.31
0.5 1.0 1.5 2.0 2.5 3.0 3.5
molar ratio

Supplementary Figure 2 ITC of Eps15-530–896 or Eps15-618–654 titrated with the SGIP1 μHD. (**a–e**) ITC of Eps15-530–896. (**a**) Baseline-corrected measured power differential (DP) as a function of time, for two independent ITC experiments (blue and red). (**b**) A nonlinear global weighted least-squares fit of heat released as a function of the added ligand. The errors arising from the baseline uncertainty were estimated with the program NITPIC[22], and are shown. The data were fitted with a 1:1 binding curve. (**c**) Residuals of the fit with a 1:1 binding curve, ranging from −0.51 to 0.79 kcal/mol. (**d**) A nonlinear global weighted least-squares fit of the same isotherm data as in **b** with a 1:2 (Eps15-618–654:μHD) binding curve. (**e**) Residuals of the fit with a 1:2 binding curve, ranging from –0.28 to 0.23 kcal/mol. (**f–j**) ITC of Eps15-618–654. (**f**) Baseline-corrected DP for two independent ITC experiments (blue and red). (**g**) A nonlinear global weighted least-squares fit of the ITC data with a 1:1 binding curve. (**h**) Residuals of the fit with a 1:1 binding curve, ranging from –0.74 to 0.95 kcal/mol. (**i**) A nonlinear global weighted least-squares fit of the same isotherm data as in **g** with a 1:2 binding curve. (**j**) Residuals of the fit with a 1:2 binding curve, ranging from –0.31 to 0.33 kcal/mol.

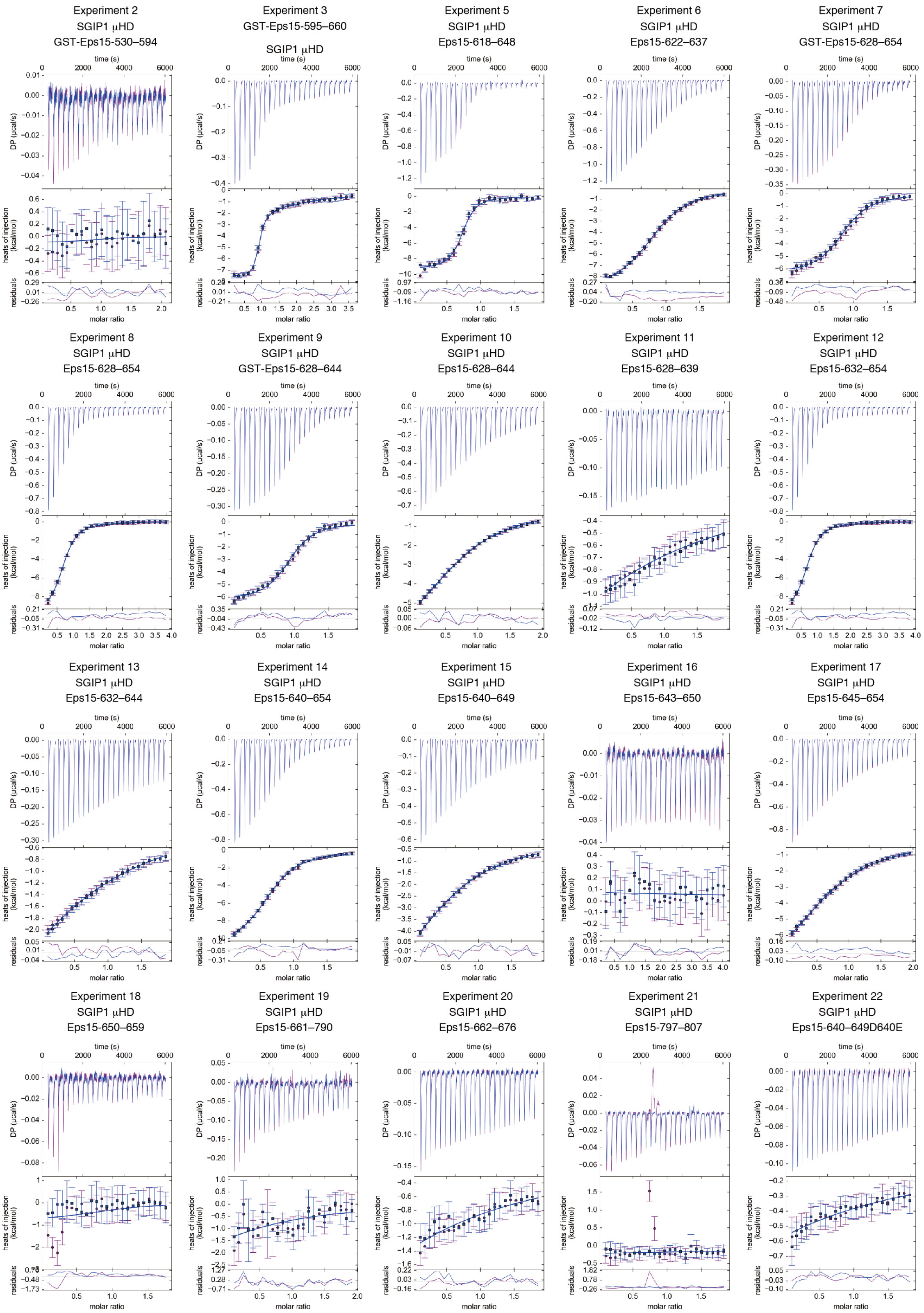

Experiment 2
SGIP1 μHD
GST-Eps15-530–594
Experiment 3
GST-Eps15-595–660
SGIP1 μHD
Experiment 5
SGIP1 μHD
Eps15-618–648
Experiment 6
SGIP1 μHD
Eps15-622–637
Experiment 7
SGIP1 μHD
GST-Eps15-628–654
Experiment 8
SGIP1 μHD
Eps15-628–654
Experiment 9
SGIP1 μHD
GST-Eps15-628–644
Experiment 10
SGIP1 μHD
Eps15-628–644
Experiment 11
SGIP1 μHD
Eps15-628–639
Experiment 12
SGIP1 μHD
Eps15-632–654
Experiment 13
SGIP1 μHD
Eps15-632–644
Experiment 14
SGIP1 μHD
Eps15-640–654
Experiment 15
SGIP1 μHD
Eps15-640–649
Experiment 16
SGIP1 μHD
Eps15-643–650
Experiment 17
SGIP1 μHD
Eps15-645–654
Experiment 18
SGIP1 μHD
Eps15-650–659
Experiment 19
SGIP1 μHD
Eps15-661–790
Experiment 20
SGIP1 μHD
Eps15-662–676
Experiment 21
SGIP1 μHD
Eps15-797–807
Experiment 22
SGIP1 μHD
Eps15-640–649D640E
time (s)
DP (μcal/s)
heats of injection (kcal/mol)
residuals
molar ratio

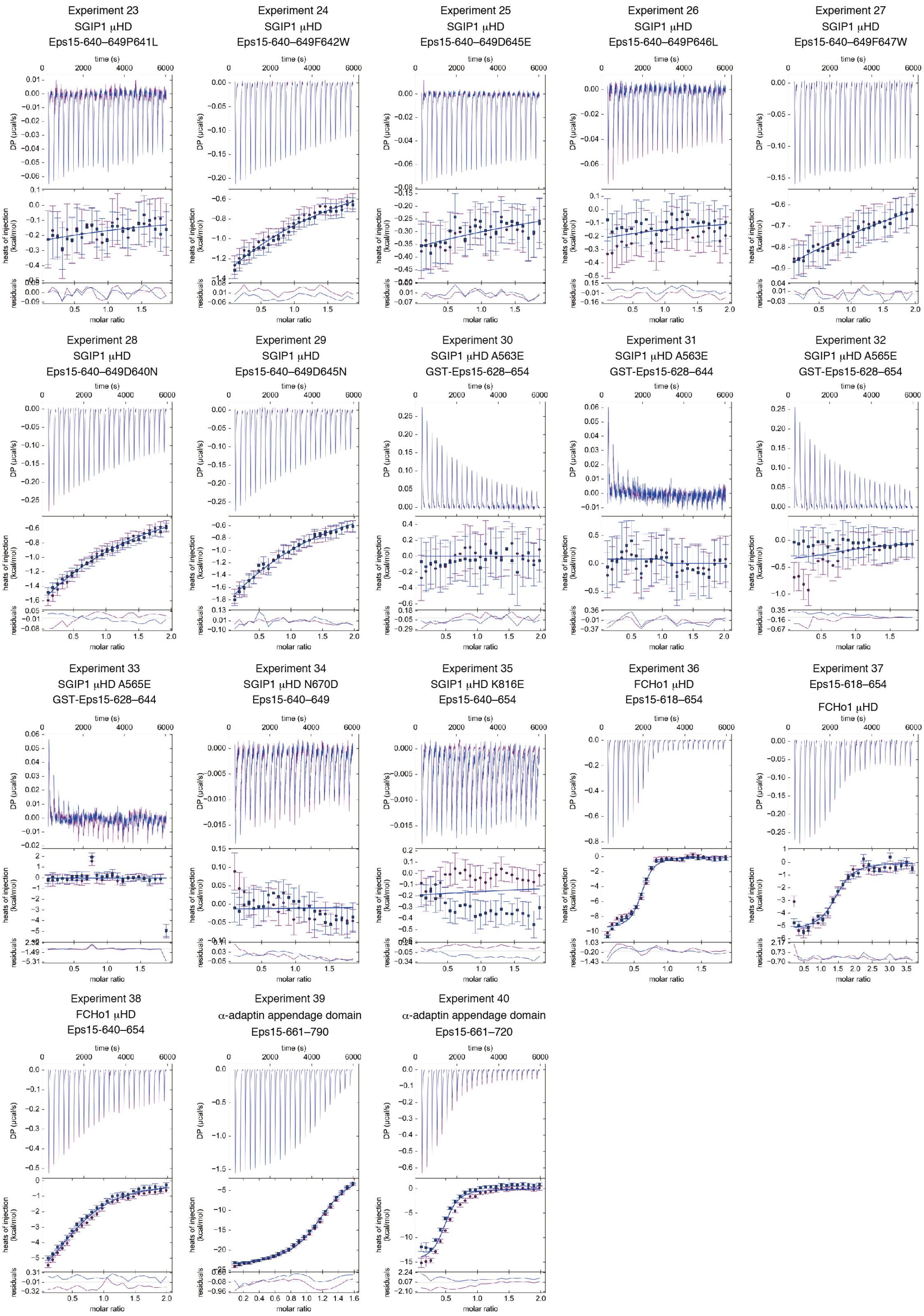
Experiment 23
SGIP1 μHD
Eps15-640–649P641L
Experiment 24
SGIP1 μHD
Eps15-640–649F642W
Experiment 25
SGIP1 μHD
Eps15-640–649D645E
Experiment 26
SGIP1 μHD
Eps15-640–649P646L
Experiment 27
SGIP1 μHD
Eps15-640–649F647W
Experiment 28
SGIP1 μHD
Eps15-640–649D640N
Experiment 29
SGIP1 μHD
Eps15-640–649D645N
Experiment 30
SGIP1 μHD A563E
GST-Eps15-628–654
Experiment 31
SGIP1 μHD A563E
GST-Eps15-628–644
Experiment 32
SGIP1 μHD A565E
GST-Eps15-628–654
Experiment 33
SGIP1 μHD A565E
GST-Eps15-628–644
Experiment 34
SGIP1 μHD N670D
Eps15-640–649
Experiment 35
SGIP1 μHD K816E
Eps15-640–654
Experiment 36
FCHo1 μHD
Eps15-618–654
Experiment 37
Eps15-618–654
FCHo1 μHD
Experiment 38
FCHo1 μHD
Eps15-640–654
Experiment 39
α-adaptin appendage domain
Eps15-661–790
Experiment 40
α-adaptin appendage domain
Eps15-661–720
time (s)
DP (μcal/s)
heats of injection (kcal/mol)
residuals
molar ratio

Supplementary Figure 3 ITC of the SGIP1/FCHo1 μHD or the α-adaptin appendage domain titrated with various Eps15 fragments, and ITC of Eps15 fragments titrated with the SGIP1/FCHo1 μHD. The data are depicted as in **Supplementary Figure 2**. The experiment numbers corresponding to those in **Supplementary Table 2**, the names of the proteins or peptides in the sample cell, and those of the titrants are shown above the corresponding ITC thermograms in the first, second, and third rows, respectively.

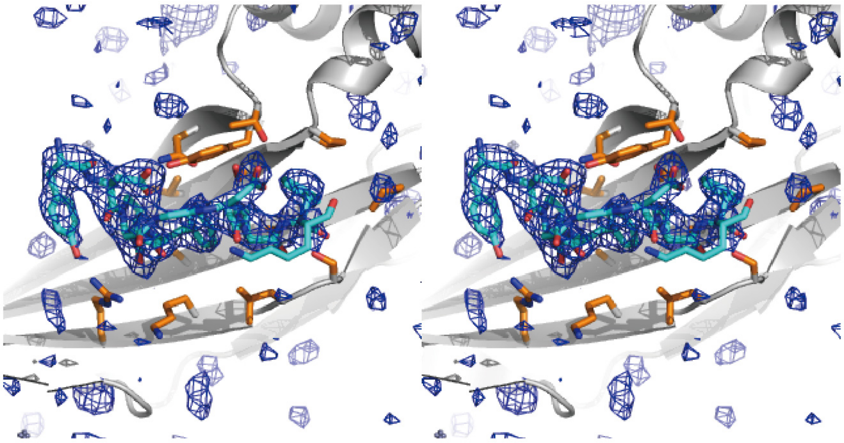

Supplementary Figure 4 Stereo view of the interaction between the μHD and Eps15-640–649 in the structure of the μHD in complex with Eps15-640–649, with a simulated annealing F_o-F_c omit electron density map contoured at +2.5σ. To create the map, Eps15-640–649 was omitted from the model. The molecules are colored as in **Figure 4c**. Eps15-640–649 and Eps15-645–654 each contain an extra (artificial) N-terminal Tyr residue, to facilitate the concentration determination by absorbance. The electron densities for this Tyr residue are clearly visible, and thus it is included in the model.

Supplementary Table 1 Amino acid sequences of Eps15 fragments used in this study.

Fragment names	Amino acid sequences
Eps15-530–896	(GPLGS)STSSSETANLNEHVEGQSNLESEPIHQESPARSSPELLPSGVTDENEVTTAVTE KVCSELDNNRHSKEEDPFNVDSSSLTGPVADTNLDFFQSDPFVGSDPFKDDPFGKIDPF GGDPFKGSDPFASDCFFRQSTDPFATSSTDPFSAANNSSITSVETLKHNDPFAPGGTVV AASDSATDPFASVFGNESFGGGFADFSTLSKVNNEDPFRSATSSSVSNVVITKNVFEET SVKSEDEPPALPPKIGTPTRPCPLPPGKRSINKLDSPDPFKLNDPFQPFPGNDSPKEKDPE IFCDPFTSATTTTNKEADPSNFANFSAYPSEEDMIEWAKRESEREEEQRLARLNQQEQE DLELAIALSKSEISEA
GST-Eps15-530–594	(**GST**[a])STSSSETANLNEHVEGQSNLESEPIHQESPARSSPELLPSGVTDENEVTTAVTEK VCSELDNNRH
GST-Eps15-595–660	(**GST**[a])SKEEDPFNVDSSSLTGPVADTNLDFFQSDPFVGSDPFKDDPFGKIDPFGGDPFKG SDPF
Eps15-618–654	(GPLGSY)DFFQSDPFVGSDPFKDDPFGKIDPFGGDPFKGSDPFA
Eps15-618–648	(GPLGSY)DFFQSDPFVGSDPFKDDPFGKIDPFGGDPFK
Eps15-622–637	(Acetyl)(Y)SDPFVGSDPFKDDPFG($CONH_2$)
GST-Eps15-628–654	(**GST**[a])SDPFKDDPFGKIDPFGGDPFKGSDPFA
Eps15-628–654	(Acetyl)(Y)SDPFKDDPFGKIDPFGGDPFKGSDPFA($CONH_2$)
GST-Eps15-628–644	(**GST**[a])SDPFKDDPFGKIDPFGG
Eps15-628–644	(Acetyl)(Y)SDPFKDDPFGKIDPFGG($CONH_2$)
Eps15-628–639	(Acetyl)(Y)SDPFKDDPFGKI($CONH_2$)
Eps15-632–654	(Acetyl)(Y)KDDPFGKIDPFGGDPFKGSDPFA($CONH_2$)
Eps15-632–644	(Acetyl)(Y)KDDPFGKIDPFGG($CONH_2$)
Eps15-640–654	(Acetyl)(Y)DPFGGDPFKGSDPFA($CONH_2$)
Eps15-640–649	(Acetyl)(Y)DPFGGDPFKG($CONH_2$)
Eps15-643–650	(Acetyl)(Y)GGDPFKGS($CONH_2$)
Eps15-645–654	(Acetyl)(Y)DPFKGSDPFA($CONH_2$)
Eps15-650–659	(Acetyl)(Y)SDPFASDCFF($CONH_2$)
Eps15-661–790	(GPLGSY)QSTDPFATSSTDPFSAANNSSITSVETLKHNDPFAPGGTVVAASDSATDPFA SVFGNESFGGGFADFSTLSKVNNEDPFRSATSSSVSNVVITKNVFEETSVKSEDEPPALP PKIGTPTRPCPLPPGKRS(W)
Eps15-662–676	(Acetyl)(Y)STDPFATSSTDPFSA($CONH_2$)
Eps15-797–807	(Acetyl)(Y)PDPFKLNDPFQ($CONH_2$)
Eps15-640–649D640E	(Acetyl)(Y)(E)PFGGDPFKG($CONH_2$)
Eps15-640–649P641L	(Acetyl)(Y)D(L)FGGDPFKG($CONH_2$)
Eps15-640–649F642W	(Acetyl)(Y)DP(W)GGDPFKG($CONH_2$)
Eps15-640–649D645E	(Acetyl)(Y)DPFGG(E)PFKG($CONH_2$)
Eps15-640–649P646L	(Acetyl)(Y)DPFGGD(L)FKG($CONH_2$)
Eps15-640–649F647W	(Acetyl)(Y)DPFGGDP(W)KG($CONH_2$)
Eps15-640–649D640N	(Acetyl)(Y)(N)PFGGDPFKG($CONH_2$)
Eps15-640–649D645N	(Acetyl)(Y)DPFGG(N)PFKG($CONH_2$)
Eps15-661–720	(GPLGSY)QSTDPFATSSTDPFSAANNSSITSVETLKHNDPFAPGGTVVAASDSATDPFA SVFGNESF

Artificial sequences and modifications are enclosed by parentheses.

[a] **GST** indicates the GST portion of the GST-fusion protein.

Supplementary Table 2 K_ds of the interactions between various Eps15-derived fragments and the SGIP1/FCHo1 μHD or the α-adaptin appendage domain determined by ITC.

Experiment numbers	Fragment names	DPF-motif numbers	Eps15 fragments (μM)	The μHD or the appendage domain (μM)	K_d (μM)	N	ΔH (cal/mol)	$-T\Delta S$ (cal/mol)	ΔG (cal/mol)
			SGIP1 μHD						
1[a]	Eps15-530–896	15	10	195	4.5 ± 2.2	0.76 ± 0.11	-10974 ± 504	3678	-7296
1[a,b]	Eps15-530–896	15	10	195	0.13 ± 0.09	0.96 ± 0.04	-5077 ± 308	-4297	-9374
	second site				27.3 ± 15.7	0.96 ± 0.04	-8970±1643	2744	-6226
2	GST-Eps15-530–594	0	207	19	below detection limit				
3[a]	GST-Eps15-595–660	7	9	173	0.046 ± 0.015	1.17 ± 0.02	-7562 ± 103	-2450	-10012
	second site				16.6 ± 4.1	1.17 ± 0.02	-6848 ± 772	328	-6520
4[a]	Eps15-618–654	6	10	191	0.42 ± 0.25	0.81 ± 0.04	-8982 ± 639	278	-8704
4[a,b]	Eps15-618–654	6	10	191	0.065 ± 0.024	0.88 ± 0.02	-7460 ± 168	-2345	-9805
	second site				55.5 (31.6~190.5)	0.88 ± 0.02	-9628 (< -6103)	3722	-5906
5	Eps15-618–648	5	288	29	0.40 ± 0.11	0.71± 0.02	-9303 ± 257	581	-8722
6	Eps15-622–637	3	506	51	4.9 ± 0.3	0.85 ± 0.01	-9214 ± 138	1967	-7247
7	GST-Eps15-628–654	5	200	21	1.0 ± 0.2	0.86 ± 0.02	-6390 ± 179	-1785	-8175
8	Eps15-628–654	5	302	15	1.1 ± 0.1	0.67 ± 0.01	-9846 ± 223	1711	-8135
9	GST-Eps15-628–644	3	162	16	0.65 ± 0.1	0.92 ± 0.02	-6285 ± 144	-2159	-8444
10	Eps15-628–644	3	509	50	20.2 ± 0.7	0.76 ± 0.01	-8019 ± 169	1615	-6404
11	Eps15-628–639	2	497	49	264.2 ± 35.9	1[c]	-6410 ± 646	1528	-4882
12	Eps15-632–654	4	497	47	3.2 ± 0.2	0.70 ± 0.01	-8944 ± 142	1453	-7491
13	Eps15-632–644	2	501	51	80.8 ± 3.3	1[c]	-5513 ± 120	-71	-5584
14	Eps15-640–654	3	302	30	2.9 ± 0.2	0.66 ± 0.01	-11041 ± 191	3482	-7559
15	Eps15-640–649	2	506	50	30.2 ± 1.8	0.66 ± 0.03	-8272 ± 679	2106	-6166
16	Eps15-643–650	1	319	15	below detection limit				
17	Eps15-645–654	2	524	51	22.4 ± 1.5	0.75 ± 0.02	-9910 ± 683	3566	-6344
18	Eps15-650–659	1	81	9	below detection limit				
19	Eps15-661–790	5	287	28	below detection limit				
20	Eps15-662–676	2	302	31	124.6 ± 28.5	1[c]	-6722 ± 1053	1395	-5327
21	Eps15-797–807	2	320	33	below detection limit				
22	Eps15-640–649D640E	1	520	54	324.7 ± 66.2	1[c]	-3908 ± 595	-851	-4759
23	Eps15-640–649P641L	1	497	50	below detection limit				
24	Eps15-640–649F642W	1	506	51	229.1 ± 24.0	1[c]	-7192 ± 527	2227	-4965
25	Eps15-640–649D645E	1	500	51	below detection limit				
26	Eps15-640–649P646L	1	492	48	below detection limit				
27	Eps15-640–649F647W	1	513	49	below detection limit				
28	Eps15-640–649D640N	1	508	50	102.8 ± 7.3	1[c]	-4856 ± 193	-585	-5441
29	Eps15-640–649D645N	1	507	48	73.1 ± 5.1	1[c]	-4504 ± 155	-1138	-5642
			SGIP1 μHD A563E						
30	GST-Eps15-628–654	5	206	20	below detection limit				
31	GST-Eps15-628–644	3	218	20	below detection limit				
			SGIP1 μHD A565E						
32	GST-Eps15-628–654	5	192	20	below detection limit				
33	GST-Eps15-628–644	3	207	20	below detection limit				
			SGIP1 μHD N670D						
34	Eps15-640–649	2	496	51	below detection limit				
			SGIP1 μHD K816E						
35	Eps15-640–654	3	201	20	below detection limit				
			FCHo1 μHD						
36	Eps15-618–654	6	206	21	0.27 ± 0.10	0.59 ± 0.02	-9638 ± 381	672	-8966
37[a]	Eps15-618–654	6	6	110	0.19 ± 0.09	0.70 ± 0.03	-7586 ± 398	-1572	-9158
38	Eps15-640–654	3	208	20	5.5 ± 1.4	0.64 ± 0.06	-7923 ± 1098	748	-7175
			α-adaptin appendage domain						
39	Eps15-661–790	5	194	23	0.69 ± 0.08	1.19 ± 0.01	-28442 ± 319	20041	-8401
40	Eps15-661–720	4	117	11	0.27 ± 0.13	0.47 ± 0.04	-14931 ± 1892	5961	-8970

N is the binding ratio of the Eps15-derived fragments to the SGIP1 μHD, the SGIP1 μHD mutants, the FCHo1 μHD, or the α-adaptin appendage domain. The data were analyzed with the single-site model, unless indicated otherwise in the table. The 68.3% confidence intervals are shown for K_d, N, and ΔH. Experiment numbers correspond to those in **Supplementary Fig. 3**.

[a] The Eps15-derived fragments were titrated with the SGIP1 μHD.

[b] The data were analyzed with the two-site model.

[c] The value for N was fixed as 1.

Supplementary Table 3 Regions of Eps15, Eps15R, and Dab2, involved in binding to the μHDs of SGIP1/ SGIP1α/FCHo1/FCHo2 from various sources.

μHD-containing protein	μHD-binding protein	Region involved in binding to the μHD	Number of DPF motifs in the binding region	Method used to confirm binding	Strength of affinity	Reference
mouse SGIP1 μHD (aa501–807)	Eps15 [a]	residues 594–896	15 [b]	pull-down assay (competition)	unknown	Reider *et al*.[8]
mouse SGIP1α μHD (aa561–854)	rat Eps15	residues 593–894	15	pull-down assay	unknown	Uezu *et al*.[19]
mouse SGIP1α μHD (aa428–854)	rat Eps15	residues 636–747	8	two-hybrid assay (consensus sequence of binding fragments)	unknown	Uezu *et al*.[19]
human SGIP1 μHD (aa552–828)	human Eps15	residues 618–654	6	ITC	high	This study
human SGIP1 μHD (aa552–828)	human Eps15	residues 662–676	2	ITC	low	This study
human FCHo1 μHD (aa610–889)	Eps15 [a]	residues 594–896	15 [b]	pull-down assay (competition)	unknown	Reider *et al*.[8]
human FCHo1 μHD (aa609–889)	Eps15 [a]	residues 595–896	15 [b]	pull-down assay	unknown	Umasankar *et al*.[23]
human full-length FCHo1	Eps15 [a]	residues 595–660	7 [b]	pull-down assay	unknown	Umasankar *et al*.[23]
human FCHo1 μHD (aa623–889)	human Eps15	residues 618–654	6	ITC	high	This study
human full-length FCHo1	Eps15 [a]	residues 595–636	4 [b]	pull-down assay	low [c]	Umasankar *et al*.[23]
mouse FCHo2 μHD (aa525–809)	human Eps15	residues 530–791	12	pull-down assay	unknown	Henne *et al*.[9]
mouse FCHo2 μHD (aa526–809)	rat Eps15	residues 593–834	15	pull-down assay	unknown	Uezu *et al*.[10]
human FCHo1 μHD (aa609–889)	human Eps15R	full length (endogenous)	21	pull-down assay	unknown	Umasankar *et al*.[23]
mouse FCHo2 μHD (aa525–809)	rat Eps15R	full length (endogenous)	21	pull-down assay	unknown	Henne *et al*.[9]
human FCHo1 μHD (aa609–889)	human Dab2	full length (endogenous)	3	pull-down assay	unknown	Umasankar *et al*.[23]
human FCHo2 μHD (aa521–810)	mouse Dab2	two consecutive DPF motifs	2	pull-down assay (mutational analysis)	unknown	Mulkearns & Cooper[11]

[a] The source from which the Eps15 is derived is not clearly stated in the reference. We assume it is human Eps15 from the total length of the protein.

[b] The number of DPF motifs is based on the assumption that human Eps15 was used.

[c] The affinity is lower than those of the other longer fragments containing more DPF motifs tested in the same reference.
